# Studying the regression profiles of cervical tumours during radiotherapy treatment using a patient-specific multiscale model

**DOI:** 10.1038/s41598-018-37155-9

**Published:** 2019-01-31

**Authors:** Christos A. Kyroudis, Dimitra D. Dionysiou, Eleni A. Kolokotroni, Georgios S. Stamatakos

**Affiliations:** In Silico Oncology and In Silico Medicine Group, Institute of Communication and Computer Systems, School of Electrical and Computer Engineering, National Technical University of Athens, Athens, Greece

## Abstract

Apart from offering insight into the biomechanisms involved in cancer, many recent mathematical modeling efforts aspire to the ultimate goal of clinical translation, wherein models are designed to be used in the future as clinical decision support systems in the patient-individualized context. Most significant challenges are the integration of multiscale biodata and the patient-specific model parameterization. A central aim of this study was the design of a clinically-relevant parameterization methodology for a patient-specific computational model of cervical cancer response to radiotherapy treatment with concomitant cisplatin, built around a tumour features-based search of the parameter space. Additionally, a methodological framework for the predictive use of the model was designed, including a scoring method to quantitatively reflect the similarity and bilateral predictive ability of any two tumours in terms of their regression profile. The methodology was applied to the datasets of eight patients. Tumour scenarios in accordance with the available longitudinal data have been determined. Predictive investigations identified three patient cases, anyone of which can be used to predict the volumetric evolution throughout therapy of the tumours of the other two with very good results. Our observations show that the presented approach is promising in quantifiably differentiating tumours with distinct regression profiles.

## Introduction

The contribution of mathematical modelling in cancer research has been ever-increasing^1,2^. The power of mathematical models lies in their ability to describe biological phenomena in the succinct language of mathematics, thereby helping to elucidate key mechanisms at play in cancer growth and response to treatment and ultimately develop predictive tools. The literature devoted to mathematical models of tumour response to therapy is vast.

Focusing on the specific case of cervical cancer response to treatment, important recent contributions include^3–6^. The main incentive for all these studies has been the fact that the regression rate of cervical tumours during radiotherapy treatment has been suggested as an important predictor of local control and long-term survival. Importantly, there is a considerable variability among patients in the regression profile of their tumours^5,6^; in some cases tumour regression is exponential as a function of time throughout treatment. In others, tumours regress more slowly early in the treatment and the relative volume plots are characterized by an initial shoulder. Huang *et al*.^6^ developed a kinetic model incorporating the radiobiological parameters of radiosensitivity, tumour repopulation, and dead cell resolving, and used it to analyze the volume regression data of 80 cervical cancer patients as assessed by serial magnetic resonance imaging (MRI). The patients were treated with external beam radiation therapy (EBRT) followed by low-dose rate intracavitary brachytherapy. 26 patients received cisplatin-based chemotherapy, which was not explicitly modelled. The analysis permitted the estimation of tumour radiosensitivity and dead cell resolving time for individual patients. Moreover, since long-term follow-up data were available, these parameters were correlated with clinical outcome.

In the study of Lim *et al*.^5^ weekly MRI was used to measure tumour volume during EBRT treatment and oxymetry with the Eppendorf electrode to assess pre-treatment tumour hypoxic fractions. The radiobiological parameters included in the mathematical model were the *in vivo* surviving proportion of cells after 2 Gy (SP_2_), the cell clearance constant (T_C_), and the cell doubling time after the onset of accelerated repopulation (T_p_). The model was fitted to the MRI-derived tumour volumes of 27 patients. The results indicate that SP_2_ and T_C_ strongly influence the shape of the volume-response curves, while SP_2_ correlates with the pretreatment measurements of hypoxia.

Similarly, Belfatto *et al*.^4^ developed a model consisting in two ordinary differential equations to study the volume regression profiles of 15 cervical cancer patients, as assessed by computed tomography imaging, and performed their analyses both on a cohort- as well as on a patient-specific base. Lately, Arnesen *et al*.^3^, utilized a previously developed mathematical model of tumour shrinkage during fractionated radiotherapy^7^ to study the regression data of 25 cervical cancer patients. The model was based on similar biological parameters as the previous described ones: the doubling time of viable cells, the half time for clearance of doomed cells and the radiosensitivity parameter α of the well-known Linear Quadratic (LQ) model. The authors additionally studied three different fractionation patterns for dose escalation.

All these studies show the value of exploiting patient specific data through the use of mathematical models for studying the biological mechanisms regulating cervical cancer response to treatment, with numerous potential applications in the future. The applied mathematical models have been kept deliberately simple, and the considered radiobiological parameters were limited, in order to facilitate the integration of imaging-derived patient-specific data and the correlation analyses. It indeed remains a significant challenge to achieve a satisfactory compromise between the need to describe more realistically the involved biological mechanisms and the goal to develop clinically relevant and personalizable mathematical models that integrate multiscale biological data^1^. The incorporation of several biological phenomena in such complex models necessitates a large number of parameters with currently unidentified or very large value ranges. This implies the need to search very large parameter spaces in order to fit the model to clinical data. The experimental or clinical measurement of several parameters may be particularly laborious or currently impossible. An intuitive grasp of the implications of the values of such low-level parameters may even be practically infeasible in the clinical setting. Such issues have generally hindered the clinical application of multiscale mathematical oncological models.

The CERvical cancer ONCOsimulator (CERONCO), developed within the context of the EU-funded DrTherapat project^8^ is a multiscale computational model of cervical cancer response to radiotherapy treatment in the patient-individualized context. The clinical orientation of the model has been a fundamental guiding principle throughout its development. CERONCO parameters are related to the explicit description of several important biological mechanisms. Central aims of this study were to clinically adapt CERONCO by exploiting sets of real multi-scale biodata, in a way that the previously described difficulties are minimized, and to demonstrate the potential of the model to offer qualitative and quantitative information on the regression profiles of cervical tumours, complementary to that of more simple radiobiological models.

For this purpose, a clinically–enhanced model parameterization methodology has been designed. The central idea of the proposed methodology lies in a tumour features-based search of the parameter space. The proposed algorithm creates a one-to-many correspondence between a number of user-selected clinically meaningful tumour features and CERONCO’s model parameter values. These features do not constitute parameters of the mathematical model; instead, based on their values the system automatically assigns adequate values to the model parameters. In this way, an interface between the clinical and the mathematical reality of the studied tumours is devised, which is expected to significantly facilitate the usage of the model and the interpretation of its results in the clinical setting.

Additionally, a methodological framework for the predictive use of the model was designed, which includes a scoring method to quantitatively reflect the similarity and bilateral predictive ability of any two tumours in terms of their regression profile. In the following sections, this framework is presented along with the results of predictive tests that have been performed at this stage of our research, by exploiting the datasets of eight cervical cancer patients.

## Materials and Methods

### Simulation model

CERONCO is a predominantly discrete multiscale computational model of cervical cancer response to treatment (external beam radiotherapy with concomitant weekly cisplatin, followed by pulsed dose rate brachytherapy) in the patient-individualized context. It makes use of the available multiscale (e.g. imaging, histological, treatment) longitudinal data of the patient. CERONCO follows a cellular automaton approach.

Core algorithms of the model can be found in our previous publications. These algorithms have been adapted and combined as necessary in order to address the particularities of the considered cervical cancer treatment. For the general features of the model see^9–11^ For basic concepts with regards to cisplatin chemotherapy modeling see^11^ and with regards to External Beam Radiation Therapy (EBRT) modeling see^9,12,13^, For related sensitivity analyses see^10,11^, A new simulation module has been developed for Pulsed Dose Rate Brachytherapy (PDR-BT) modeling. This module is presented below, along with a basic outline of the main simulation model, to facilitate the understanding of the current work.

The region of interest (Gross Tumour Volume, GTV) as derived from the imaging data is represented by a three-dimensional discretization mesh. The elementary volume of the mesh is called Geometrical Cell (GC). At initialization, each GC accommodates a number of biological cells (defined based on the typical cell density of 10^9^ cells/cm^3^, unless more specific information for a particular tumour is available); each cell can belong in any of the following five classes: Stem cell; LIMP cell (LImited Mitotic Potential or committed progenitor cell); Terminally differentiated cell; Apoptotic cell; Necrotic cell. The cell cycle phases (G1, S, G2, M) and the dormant (G0) phase constitute subclasses in which stem or LIMP cells may reside.

**Figure 1:**
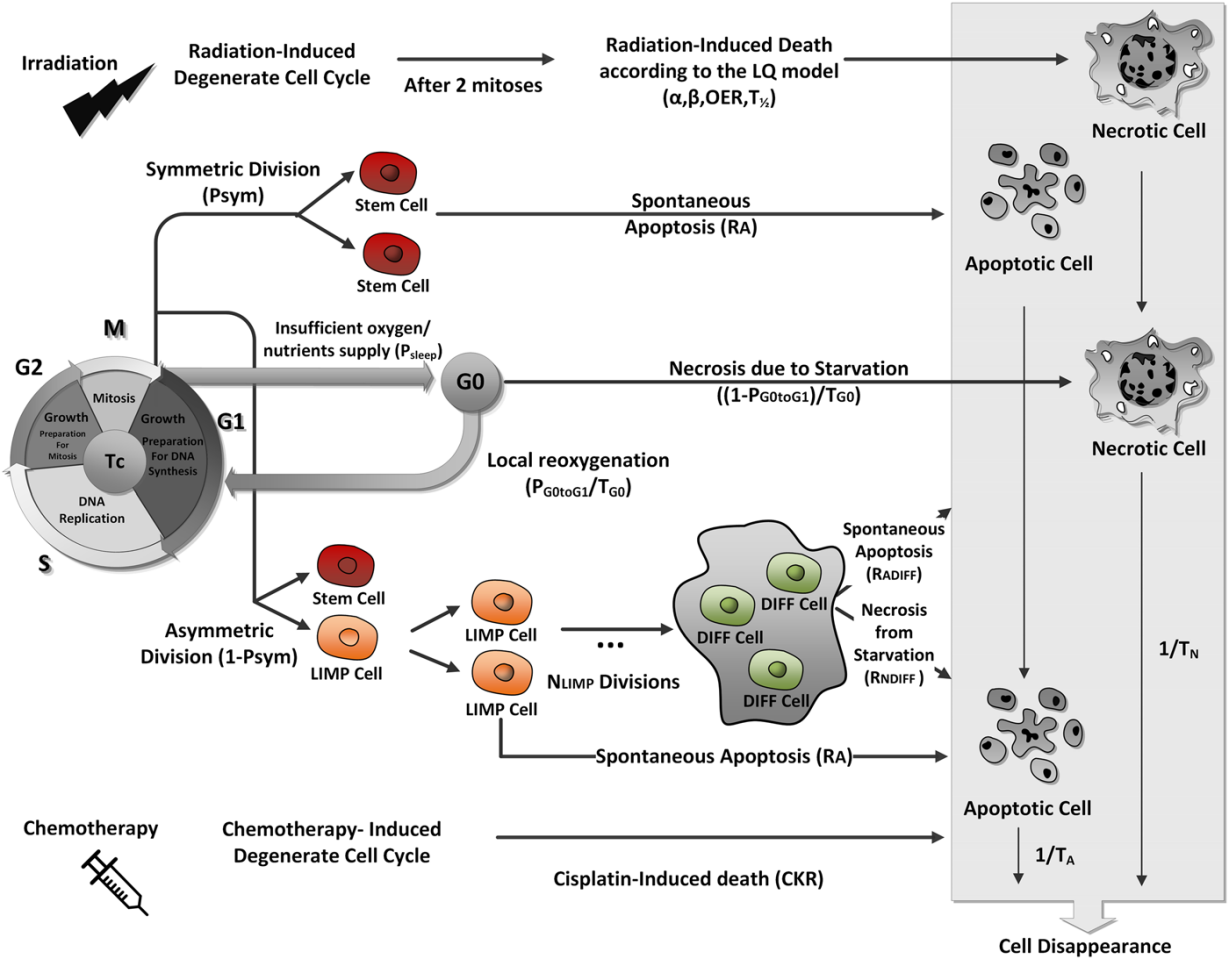
Schematic depiction of CERONCO’s cytokinetic decision calculator. The calculator dictates the transitions between cell states, which are determined by corresponding model parameters (see Table 1) with a time step of 1 h. It incorporates: cycling of proliferating cells through the successive cell cycle phases; symmetric and asymmetric modes of stem cell division; terminal differentiation of committed progenitor cells after a number of mitotic divisions; transition of proliferating cells to the dormant phase due to inadequate supply of oxygen and nutrients; reentering of dormant G0 cells into the active cell cycle due to local restoration of oxygen and nutrient supplies, spontaneous apoptosis, necrosis of inadequately nourished tumour cells, irradiation-induced cell death through necrosis, chemotherapy induced cell death through apoptosis. LIMP: Limited Mitotic Potential cells. DIFF: Terminally differentiated cells. G1: Gap 1 phase. S: DNA synthesis phase. G2: Gap 2 phase. M: Mitosis. G0: dormant phase. Hit: cells lethally hit by irradiation/drug.

Figure 1 depicts CERONCO’s cytokinetic decision calculator, which dictates the transitions between cell states (1-hour time step). The cytokinetic model incorporates several cellular-level phenomena: cycling of proliferating cells, symmetric and asymmetric stem cell division, terminal differentiation of LIMP cells, transition of proliferating cells to dormancy, reentrance of dormant hypoxic cells into the active cell cycle, necrosis of inadequately nourished tumour cells, spontaneous apoptosis, and cell necrosis and apoptosis due to therapy. Cell kill by EBRT is modelled based on the Linear-Quadratic (LQ) model^9,12,13^ Additive toxicity of radiotherapy and chemotherapy is considered^14^. The model offers the possibility of assigning increased radiosensitivity/chemosensitivity to stem cells compared to LIMP cells^15–17^.

A new module simulating PDR-BT treatment has been developed, based on the modified LQ model with correction for incomplete repair^17–20^. Considering a fraction of PDR-BT consisting of N pulses of dose d and an inter-pulse interval on the order of one hour, sub-lethal damage may not be completely repaired and the final survival fraction is given by:

1 $${{\rm{S}}{\rm{F}}}_{{\rm{N}}}({\rm{d}})=\exp [{\textstyle \text{-}}(\alpha {\rm{N}}{\rm{d}}+{\beta {\rm{N}}{\rm{G}}}_{{\rm{N}}}{{\rm{d}}}^{2})]$$

where G_N_ is the Lea-Catcheside factor, and the α (alpha) and β (beta) parameters are the linear and the quadratic radiosensitivity coefficients, respectively, of the irradiated cells.

The LQ model is based on the curvilinear nature of dose-response curves for the log of cell survival^21^. It considers two cell-killing components: a linear component (alpha component) and a quadratic component (beta component). The parameters alpha and beta determine the initial slope and the degree of downward curvature, respectively, of the survival curve. According to the most common mechanistic interpretation, the yield of lethal lesions is the sum of lethal lesions produced from single radiation tracks (which are linearly related to dose, the alpha component) and lethal lesions produced from two radiation tracks (which are quadratically related to dose, the beta component); i.e. the latter quantifies the interaction of sublethal events. The dose at which these two components of cell killing are equal is the alpha/beta ratio. Since sublethal lesions can be repaired prior to resulting in a lethal event, the beta component is modified by the Lea-Catcheside time factor (G_N_) to take into account dependence on dose protraction or fractionation^21^. Protracting the exposure time potentially allows the first lesion to be repaired before the second is produced, and the LQ approach quantifies this effect^21^. In general, G_N_ is determined by the rate of sublethal damage repair and the particular fractionation pattern with which the dose is delivered. It is a dimensionless quantity that can take values from 0 to 1. For acute exposures ${G}_{N}\to 1$, and for very long exposures ${G}_{N}\to 0$ (“acute” and “long” are defined relative to the half -time for sublethal damage repair).

Several studies addressing the issue of G_N_ factor calculation for PDR-BT appear in literature^19,22^, The computation of the G_N_ factor is based on the temporal characteristics of the dose (number of pulses, N, pulse duration, t, inter-pulse interval, x,) and the irradiated cells’ repair half-time T_1/2_. Repair is assumed to follow first-order kinetics and is modelled by a monoexponential function with rate constant μ (see also equation (7) below). The derivation of the following equation, used within CERONCO after each successive pulse to compute the survival fraction, is presented in the Supplementary Material (Section SA):

2 $$S{F}_{i}(d)=\exp (-\alpha d)\exp [-\beta {d}^{2}(i{G}_{i}-(i-1){G}_{i-1})],\,{\rm{i}}=1,\,\ldots ,\,{\rm{N}}$$

G_i_ is computed $\,\forall \,i\ge 2$ by the equations:

3 $${G}_{i}(PDR)=\frac{2}{\mu t}[1-\frac{iY-S{Y}^{2}}{i\mu t}]$$

4 $$Y=1-{e}^{-\mu t}$$

5 $$S=\frac{iK-K-i{K}^{2}{e}^{-\mu t}+{K}^{i+1}{e}^{-\mu it}}{{(1-K{e}^{-\mu t})}^{2}}$$

6 $$K={e}^{-\mu x}$$

7 $$\mu =\frac{ln2}{{T}_{1/2}}$$

where t is the duration of each pulse, x is the time between pulses without irradiation, μ is the repair rate constant, and T_1/2_ is the half time for sub-lethal damage repair.

For the first pulse the following equation is used^23^:

8 $${G}_{1}=\frac{2\,[\exp (-\mu t)+\mu t-1]}{{(\mu t)}^{2}}$$

Equation (8) can be derived from equations (3–7) for i = 1 and x → ∞.

Equation (2) is a modification of the LQ model that can be used after each successive pulse and takes into account the current number of living tumour cells. This number is defined by the competing processes of cell death (due to radiotherapy, apoptosis, and necrosis) and cell birth as incorporated in the cytokinetic model of Fig. 1.

Following tumour initialization (section “The tumour profile concept”), at each subsequent time step the mesh is scanned and the spatiotemporal evolution rules are applied. Each complete scan can be viewed as consisting of two sequential scans^10^. The first one updates the state of each GC by applying the rules of the cytokinetic model of Fig. 1. The second one deals with the rules governing the movement of cells throughout the tumour region. Τhe non-uniform dose distribution of BT renders a spatial handling of the tumours imperative.

A concise description of CERONCO tumour dynamics parameters is given in Table 1. A literature review has been performed to retrieve typical parameter values and value ranges for cervical cancer tumours. Table 1 includes important literature-derived quantitative information about CERONCO parameters and other tumour features whose values result from the selection of model parameter values.

**Table 1:** CERONCO tumour dynamics parameters and derived-therefrom tumour features, along with literature-defined typical values and plausible value ranges. Citations to indicative articles are provided. “Typical values” (when available) refer to the most commonly used values in the relevant literature, and usually (but not in all studies) correspond to mean values.

Model parameter	Description	Literature-based approximate value range, Typical values	Indicative references
T_c_ (h)	Cell cycle duration	16–70 Typical: 24–26	^41– 43^
T_G0_ (h)	G0 duration	96–240	^25, 44^
T_N_ (h)	Necrosis duration	34–3456	^5, 6, 45, 46^
T_A_ (h)	Apoptosis duration	0–25	^47^
N_LIMP_	Number of LIMP (Limited Mitotic Potential) cell mitoses before terminal differentiation	Up to 18	^46, 48^
α (Gy^−1^)	Alpha parameter of the LQ model	0.01–0.7 Typical: 0.3	^6, 14, 19, 41, 49^
β (Gy^−2^)	Beta parameter of the LQ model	0.001–0.06 Typical α/β = 10 Gy	^49, 50^
OER	Oxygen Enhancement Ratio	1.5–3.0 Typical: 2	^14, 23^
T_1/2_ (h)	Sublethal damage repair half-time	0.26–5.7 Typical: 1.5	^19, 22, 23, 49, 50^
CKR cisplatin	Cisplatin Cell Kill Rate (fraction of stem and LIMP cells lethally hit at each drug administration)	0–0.86	^14, 15, 51^
R_A_ (h^−1^)	Spontaneous apoptosis rate of stem and LIMP cells (fraction of stem and LIMP cells dying through spontaneous apoptosis per hour)	0.0004–0.008	^48^
R_NDiff_ (h^−1^)	Necrosis rate of differentiated cells (fraction of differentiated cells dying through necrosis per hour)	—	—
R_ADiff_ (h^−1^)	Spontaneous apoptosis rate of differentiated cells (fraction of differentiated cells dying through spontaneous apoptosis per hour)	0.09–0.104	^48^
P_G0toG1_ (h^−1^)	Fraction of dormant cells that re-enter cell cycle per hour	0.01–0.06	
P_sleep_	Fraction of stem and LIMP cells entering G0 phase after mitosis	0.0–1.0	^11, 44^
P_sym_	Fraction of stem cells at mitosis that perform symmetric division	0.2–0.66	^11, 46^
**Tumour characteristic**	**Literature-based approximate value range, Typical values**		
Tumour Volume Doubling Time T_d_ (days)	20–3750 Typical: 80–300		^35, 36, 42^
Growth fraction GF (percentage of proliferating tumour cells over total living tumour cells) (%)	4.1–97.8% Typical: 40–50%		^26– 28^
Hypoxic fraction (percentage of hypoxic tumour cells over total tumour cells) (%)	0–99.2% Typical: 30–60%		^5, 25, 26, 29, 30^
Apoptotic fraction (percentage of apoptotic tumour cells over all living tumour cells) (%)	0–6.8% Typical: 1%		^27, 28, 51^
Stem cell fraction (percentage of tumour stem cells over all living tumour cells) (%)	0.13–7% Typical: 1%		^16, 17, 52– 54^

### Patient data

Eight patients with squamous cervical carcinoma have been included in our study (Supplementary Material Section SB.1). The patients were treated as part of the EMBRACE clinical study^24^. The therapeutic protocol involves EBRT with concomitant cisplatin, followed by two PDR-BT fractions. Follow-up data were not available.

The patient-specific imaging data included T2 weighted MRI-derived 3D reconstructions of the Gross Tumour Volume for up to five time points:

- Pretherapy (before start of EBRT)
- Midterm (during EBRT)
- BT0 (before start of BT)
- BT1 (start of first BT fraction)
- BT2 (start of second BT fraction)

These 3D-reconstruction files supply the model with the tumour’s spatial information and correspond to the region of interest onto which the discretizing mesh is superimposed. Each GC of the mesh is labeled as tumour or non/tumour. BT1 and BT2 spatial dose distribution files (total dose per GC) are provided as well. For a short outline of the procedure used to create the above files see Supplementary Material (Section SB.2).

Patient-specific treatment data included:

- EBRT schedule: total dose, number of fractions, fractionation scheme (dates for the 5 fractions per week − 1 fraction per day, no irradiation during weekends)
- Cisplatin administration schedule (number of cycles − once per week)
- PDR-BT schedule (two fractions of 20 pulses each, inter-pulse interval, pulse duration, date of each fraction administration). The GC pulse dose is derived from the total dose distribution file, by dividing the total dose to the GC by the number of pulses. The interval between successive pulses is 1 hour and the pulse duration is variable (0.2–0.3 hours).

All patient information was given in the context of the EU-FP7 project DrTherapat, Grant agreement no. 600852. Patient information was obtained with due observance of the rights of all patients involved and in compliance with all applicable laws and regulations, including the Declaration of Helsinki as revised by the World Medical Assembly, as well as the applicable procedures and the internal guidelines of the institution. Prior to the disclosure of the patient information the clinical institution has obtained appropriate informed consents from all the patients involved, or approval from the applicable ethical review board has been obtained, all in compliance with Applicable Patient Regulations. The patients were accrued at Aarhus University Hospital and the name of the ethical committee is “Videnskabsetisk komité, Region Midt, Denmark”.

#### The tumour profile concept

A new virtual tumour initialization and parameter estimation workflow has been designed.

The first step is to assign values to the following three salient characteristics of the initial tumour, hereafter called **“the tumour profile”**:

1. Growth Fraction
  (GF, percentage of proliferating cells over all living tumour cells)
2. Hypoxic Fraction
  (HF, percentage of hypoxic cells, residing outside of the active cell cycle, over all tumour cells)
3. Dead Fraction
  (DF, percentage of dead cells over all tumour cells)
  Additionally, the user selects:
4. A cisplatin cell kill rate (CKR) value, reflecting the chemosensitivity of cancer cells to cisplatin
5. A tumour volume doubling time (T_d_) value or a series of T_d_ values.

Subsequently, the algorithm automatically determines sets of model parameter values that conform to these initial tumour characteristics. There exists a one-to-many correspondence between these features {GF, HF, DF, Td, CKR} and possible sets of model parameter values. In the present study, the algorithm determines 100 sets of parameter values; it excludes parameter values lying outside the acceptable value ranges. For the mathematical relationships between tumour features and model parameter values see^11^ and Supplementary Section SC.

In this way, different sets of CERONCO parameter values can be grouped to different solution families, defined by the selected values of tumour features. This simulation workflow creates in essence an interface between the clinical and the mathematical reality of tumour evolution.

The tumour profile-based search can consider various regions of the parameter space which imply different tumour behaviour. Different profiles translate into different initial tumour constitutions in terms of the various cell populations (proliferating cells, dormant cells etc.), which in turn are expected to exhibit variable response to therapy and long-term evolution.

If the user cannot acquire tangible patient-specific information about the tumour features, then they can test candidate scenarios. Otherwise, the available data should be used in order to refine the initialization procedure. GF and HF estimation in cervical cancer has been intensely researched, e.g. through Ki-67 studies and polarographic electrodes, respectively^5,25–30^, Recently, imaging-based methods have been also reported^31–34^. DF estimation can be based on MRI data, as was the case in our study. Similarly, literature abounds with methods for estimation of tumour volume doubling time^35,36^.

In sharp contrast, information about cisplatin chemosensitivity of cervical cancer cells is rather scarce; some efforts have addressed this issue as well^14–16^. Previous model sensitivity analyses indicate that this tumour feature has a profound impact on the result of therapy^37^. We have therefore decided to expose this crucial tumour characteristic as an adjustable feature of a simulation, in order to offer the possibility of testing several explicit scenarios.

Following initialization, tumour evolution is simulated according to the patient-specific treatment data. When the simulation is complete, the algorithm checks whether there is longitudinal volumetric agreement between the simulated and the clinical tumour, taking into account possible tumour delineation errors. A solution is a set of model parameter values for which the simulated tumour’s Volume Reduction Percentage (VRP)

9 $$VR{P}_{sim}=[\frac{{V}_{initial}^{sim}-\,{V}_{final}^{sim}}{{V}_{initial}^{sim}}]\ast 100 \% $$

differs up to a predefined threshold from the corresponding VRP_clin_, the latter calculated based on the real tumour’s GTV data, at all timepoints for which volumetric data are available. ${V}_{initial}^{sim}$ and ${V}_{final}^{sim}$ are the simulated initial and final tumour volume, respectively. The initial tumour volume is the pre-therapy volume. The final tumour volume can correspond to any of the subsequent time-points for which GTV is available.

The deviation thresholds between the clinical and simulated tumour VRPs have been chosen so as to reflect the existence of the abovementioned possible tumour contouring errors. Since the exact magnitude of such errors is unknown^38^, several different criteria for volumetric compliance were tested:

10 $$\bullet \,{\bf{V}}{\bf{R}}{\bf{P}}\,{\bf{5}}:|VR{P}_{simulated}-{\mathrm{VRP}}_{{\rm{clinical}}}|\le \,5 \% ,\,\mathrm{for}\,\mathrm{Midterm},{\rm{BT}}0,{\rm{BT}}1,{\rm{BT}}2\,$$

11 $$\bullet \,{\bf{V}}{\bf{R}}{\bf{P}}\,{\bf{10}}:\,|VR{P}_{simulated}-{{\rm{VRP}}}_{{\rm{clinical}}}|\le 10 \% ,\,{\rm{for}}\,{\rm{Midterm}},\,{\rm{BT}}0,\,{\rm{BT}}1,\,{\rm{BT}}2\,$$

12 $$\begin{array}{c}\bullet \,{\bf{M}}{\bf{i}}{\bf{x}}{\bf{e}}{\bf{d}}:\,|VR{P}_{simulated}\,-\,{{\rm{VRP}}}_{{\rm{clinical}}}|\le \,5 \% ,\,{\rm{for}}\,{\rm{Midterm}},\,{\rm{BT}}0\\ \,and\,|VR{P}_{simulated}\,-\,{{\rm{VRP}}}_{{\rm{clinical}}}|\le 10 \% ,\,{\rm{for}}\,{\rm{BT}}1,\,{\rm{BT}}2\end{array}$$

13 $$\begin{array}{c}\bullet \,{\bf{40}} \% \,{\bf{v}}{\bf{o}}{\bf{l}}{\bf{u}}{\bf{m}}{\bf{e}}\,{\bf{d}}{\bf{e}}{\bf{v}}{\bf{i}}{\bf{a}}{\bf{t}}{\bf{i}}{\bf{o}}{\bf{n}}({\bf{40}} \% \,{\bf{d}}{\bf{V}}):\\ \,|GT{V}_{simulated}-GT{V}_{clinical}|\le 0.4\ast GT{V}_{clinical},\,{\rm{for}}\,{\rm{Midterm}},\,{\rm{BT}}0,\,{\rm{BT}}1,{\rm{BT}}2\end{array}$$

The last criterion has been based on clinical experience with regards to delineation errors for the case of cervical cancer^38^. Depending on the specific value of a tumour’s volume at a particular timepoint, this criterion may be stricter or more lenient than VRP 10.

The described workflow ensures a multi-level compliance of the virtual tumour with: (a) the predefined tumour features, (b) longitudinal volumetric data, (c) any tumour characteristics for which clinical data are available (e.g. in the studied cases, the diameter of the necrotic component of the initial tumour, which dictates the tumour’s DF feature), and (d) biologically plausible value ranges of the model parameters and the derived therefrom tumour characteristics. These have been retrieved from literature for the specific tumour histological type whenever possible.

The use of a performance criterion serves for quantitatively representing the agreement of a derived solution with the real volumetric data; the error-measure Mean Absolute Error (MAE) was chosen^39^:

14 $$MA{E}_{solution}=\frac{{\sum }_{i}|VR{P}_{clinical}-VR{P}_{simulated}|}{N},\,i=\{Midterm,\,BT0,\,BT1,\,BT2\}\,( \% )$$

N: the number of timepoints with tumour volumetric data.

Solutions with lower MAE values imply better agreement with the clinical data.

#### Predictive use of the model: a new methodological framework Mean value parameter sets: assigning a single representative parameter value set to each patient/tumour profile/CKR value combination

For each patient, and for each tumour profile and CKR value, the parameter estimation algorithm typically identifies a large number of solutions with tumour doubling times within the acceptable value range, which all belong to a particular solution family. We have observed that it is possible to use the members of a specific solution family in order to identify a single parameter value set that could be assigned to a specific patient, tumour profile, and CKR-value combination. This characteristic set is created by assigning to each parameter the mean of the corresponding values in the solution family.

New simulation runs have been performed using these mean value parameter sets. Their performance in terms of volumetric agreement with the real tumour, and therefore their effectiveness in representing a particular tumour profile of a patient, can be quantified by using the Mean Absolute Error (MAE) of equation (14):

15 $$\begin{array}{rcl}MA{E}_{patient,tumourprofile,CKRvalue} & = & \frac{{\sum }_{i}|VR{P}_{clinical}-VR{P}_{mean \mbox{-} value \mbox{-} set}|\,}{N}\,\,( \% )\\ i & = & \{Midterm,\,BT0,\,BT1,\,BT2\}\end{array}$$

where: N: the number of timepoints with tumour volumetric data, VRP_mean-value-set_: the VRP for the simulated mean value parameter tumour.

The mean value derived from equation (15) for all CKR values characterizes a specific patient and tumour profile:

16 $$MA{E}_{patient,tumourprofile}=({\sum }_{i}^{N}\,MA{E}_{patient,tumourprofile,CKRvalue})/M\,( \% )$$

where M is the number of distinct CKR values having retrieved adaptation solutions.

**Figure 2:**
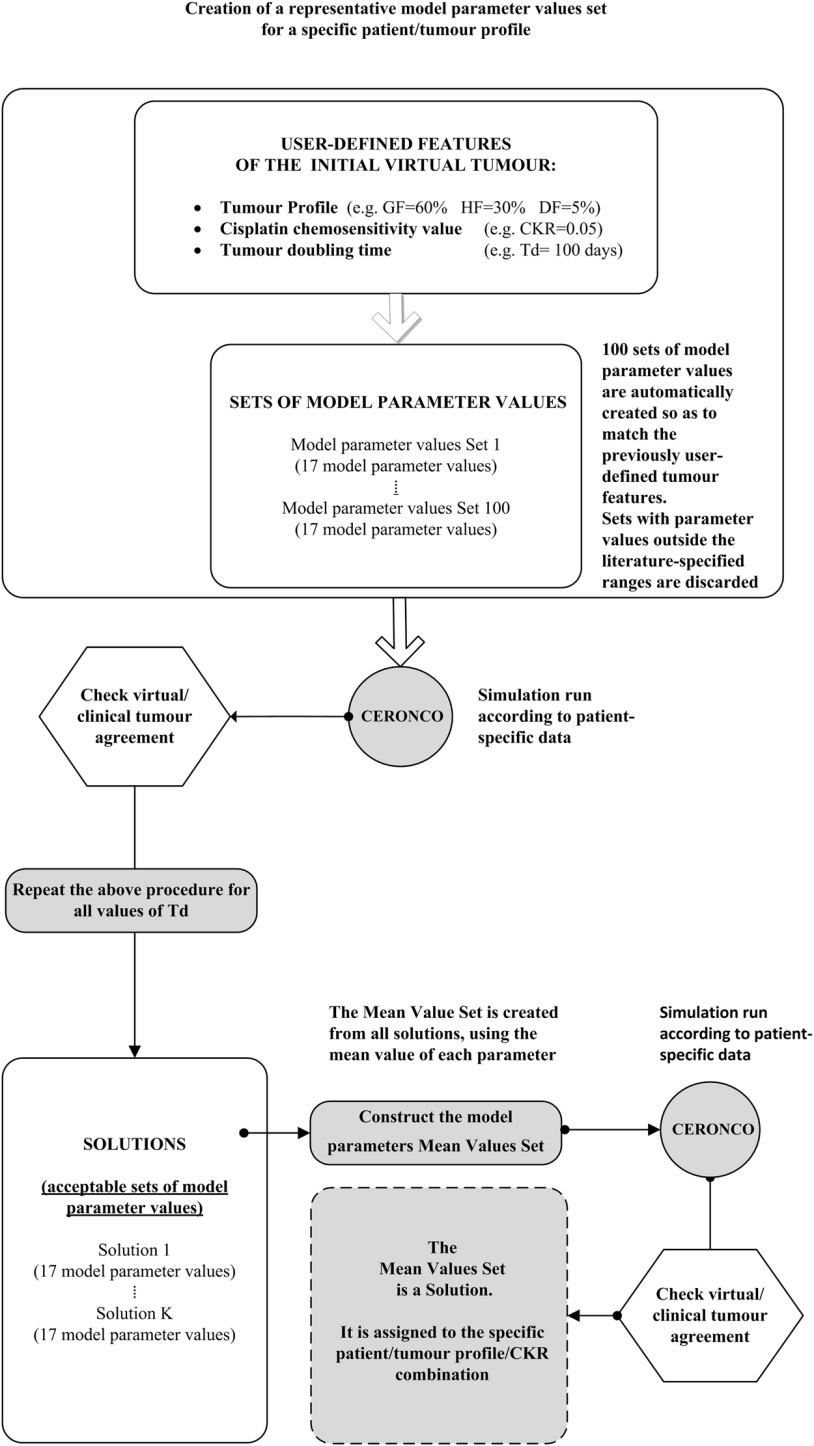
Simplified flowchart of the creation of mean value parameter sets for a specific tumour profile.

Figure 2 is a flowchart outlining the creation of the mean value parameter sets.

#### Predictive tests: patient pair methodology

Since the number of patients was limited, a formal evaluation of the predictive ability of CERONCO was out of the scope of our work. Nevertheless, a set of predictive tests has been performed. These tests are based on the consideration of patient pairs, wherein the mean value set assigned to a specific patient and tumour profile is used to predict the evolution of another patient’s tumour for the same tumour profile (and vice versa).

By running simulations for patient B, Profile I, and each CKR value separately, using the corresponding mean value parameter sets of patient A, we can compute the $MA{E}_{B\leftarrow A}$ error using equation 16. Similarly, an $MA{E}_{A\leftarrow B}$ error characterizes the handling of patient A using patient B parameter values. A total error can be assigned to the “A and B clinical case pair”:

17 $$MA{E}_{A\leftrightarrow B}=\frac{MA{E}_{A\leftarrow B}\,+\,MA{E}_{B\leftarrow A}}{2}\,( \% )$$

When for a particular profile only one of the two clinical cases of a pair has retrieved solutions, then the MAE value of the single available run is assigned to the pair error. This simplification involves 2 out of the 8 patients, and has been adopted in order to preserve the generality of the pair methodology.

We can expect that the lower MAE_A<−>B_ is, the higher is the similarity in volumetric regression terms between A and B and the bilateral predictive ability of A and B. As exemplified in the following sections, through the pair methodology the similarity of the regression profiles of any two tumours is reflected quantitatively in their $MA{E}_{A\leftrightarrow B}$ values.

## Results

Two tumour profiles have been studied for each patient: {GF = 60%, HF = 30%, DF = 5%} and {GF = 10%, HF = 30%, DF = 5%}. These are two characteristically different regions of the profile space and produce tumours with very different behaviour (a high proliferative vs a low proliferative one). The dead fraction was kept low, since the tumours had no initial necrotic components. The only exception was patient 71, but in this case too the MRI-calculated initial necrotic diameter is very small compared to the entire tumour’s equivalent diameter, and is well in agreement with a DF value of 5%. Since necrotic regions are typically associated with hypoxic regions^40^ we have considered a relatively low HF value. The cell kill rate of cisplatin has been tested from very low to rather large values (0.0–0.5). The tumour volume doubling times cover the entire value range reported in literature, with an increment of 20 days in the lower T_d_ region, up to 500 days, and an increment of 500 days subsequently.

Supplementary Table S3 presents the number of solutions identified for each profile. The results indicate the potential of CERONCO to distinguish between tumour profiles that are compatible with the actual evolution of a clinical tumour from others that are incompatible. For example, for patient 71, only the low proliferative profile retrieves solutions, whereas the reverse is true for patient 88. Some patient cases acquire solutions for the whole range of CKR values tested (e.g. 68, 71, 86), whereas others only for a subset thereof (e.g. 50, 55, 77).

Each solution represents a distinct tumour scenario compatible with the data of a patient. Different solutions imply different constitutions of the initial tumour in terms of the various tumour cell populations. These in turn result to variable post-therapy tumour constitutions, which are expected to display variable tumour regrowth potential. Such a characteristic example is presented in Supplementary Fig. S2.This observation shows that agreement with clinical data in volumetric terms alone may mask tumours with radically different characteristics and, hence, prognosis.

Supplementary Tables S4–S12 present the value ranges of CERONCO model parameters for each patient for the low proliferative profile. The corresponding information for the high proliferative profile is given in Supplementary Tables S13–S21. In Supplementary Tables S12 and S21 the range of the mean value of each parameter across all tested CKR values is presented for the two profiles. For some cases the retrieved solutions cover the entire range of volume doubling times (e.g. 50, 68, 86 for the low proliferative profile), whereas in other cases the solutions reside in subsets thereof (e.g. all patients for the high proliferative profile). Similar observations can be made with regards to the values of the various model parameters in comparison with the literature-derived value ranges. The assignment of values or possible value ranges to the model parameters, under all the constraints imposed by the available data, complements the characterization of a tumour in terms of those features for which experimental/clinical information is lacking.

**Figure 3:**
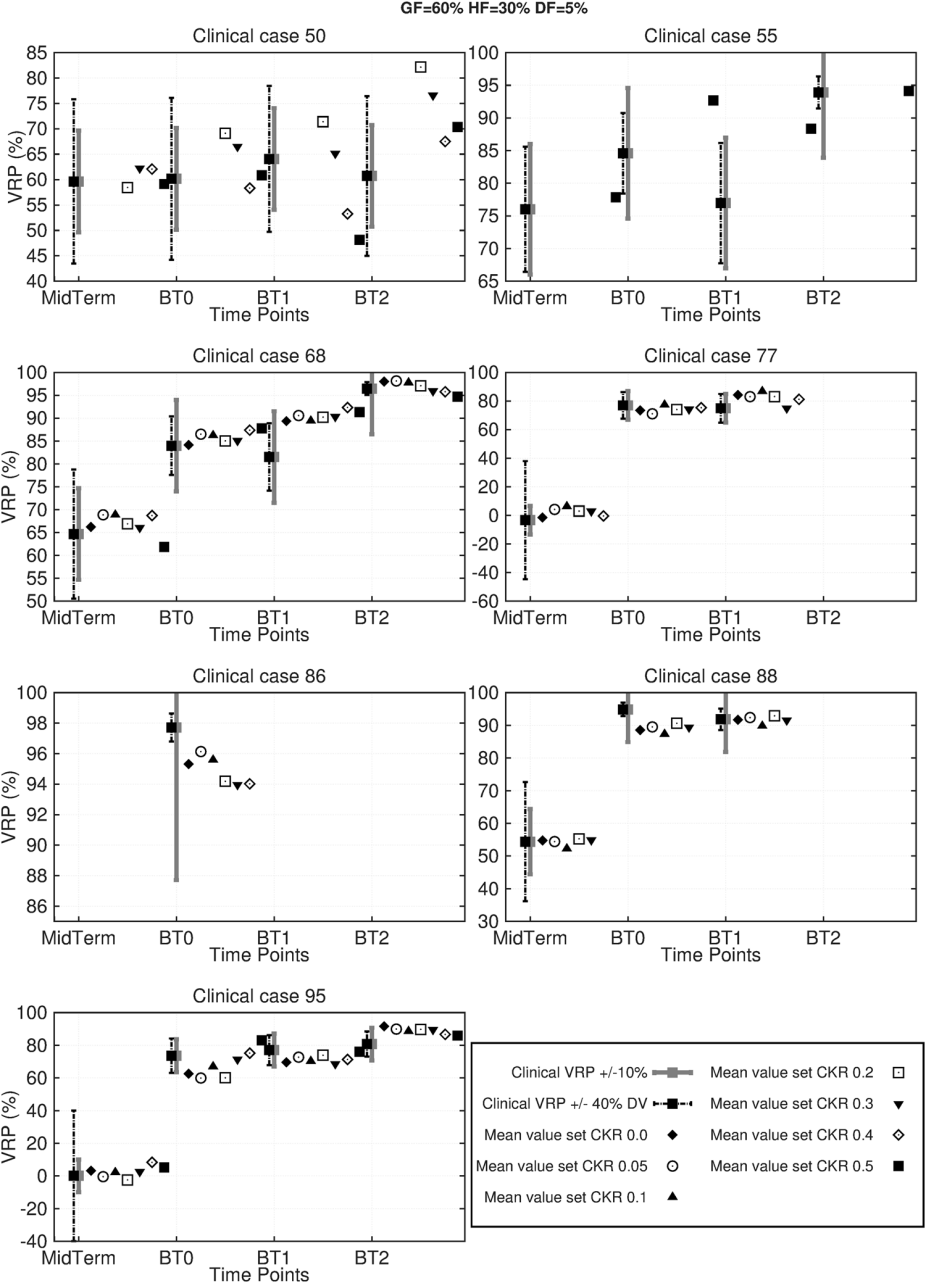
Simulations with mean value parameter sets for the high proliferative tumour profile. Comparison of clinical and simulated Volume Reduction Percentages (VRP), for the tumour profile GF = 60%, HF = 30%, DF = 5%. The simulated VRP at each time-point is compared to the corresponding clinical VRP. The error bars indicate the permitted variability around the clinical VRP value according to the criteria VRP 10 (equation 11) and 40% DV (equation (13)). This variability is introduced to reflect possible tumour delineation errors. VRP values are computed according to equation (9). Note: For each timepoint (midterm, BT0, BT1, BT2) the VRPs for each CKR value are horizontally displaced to facilitate the inspection of the results; this displacement does not have temporal meaning.

**Figure 4:**
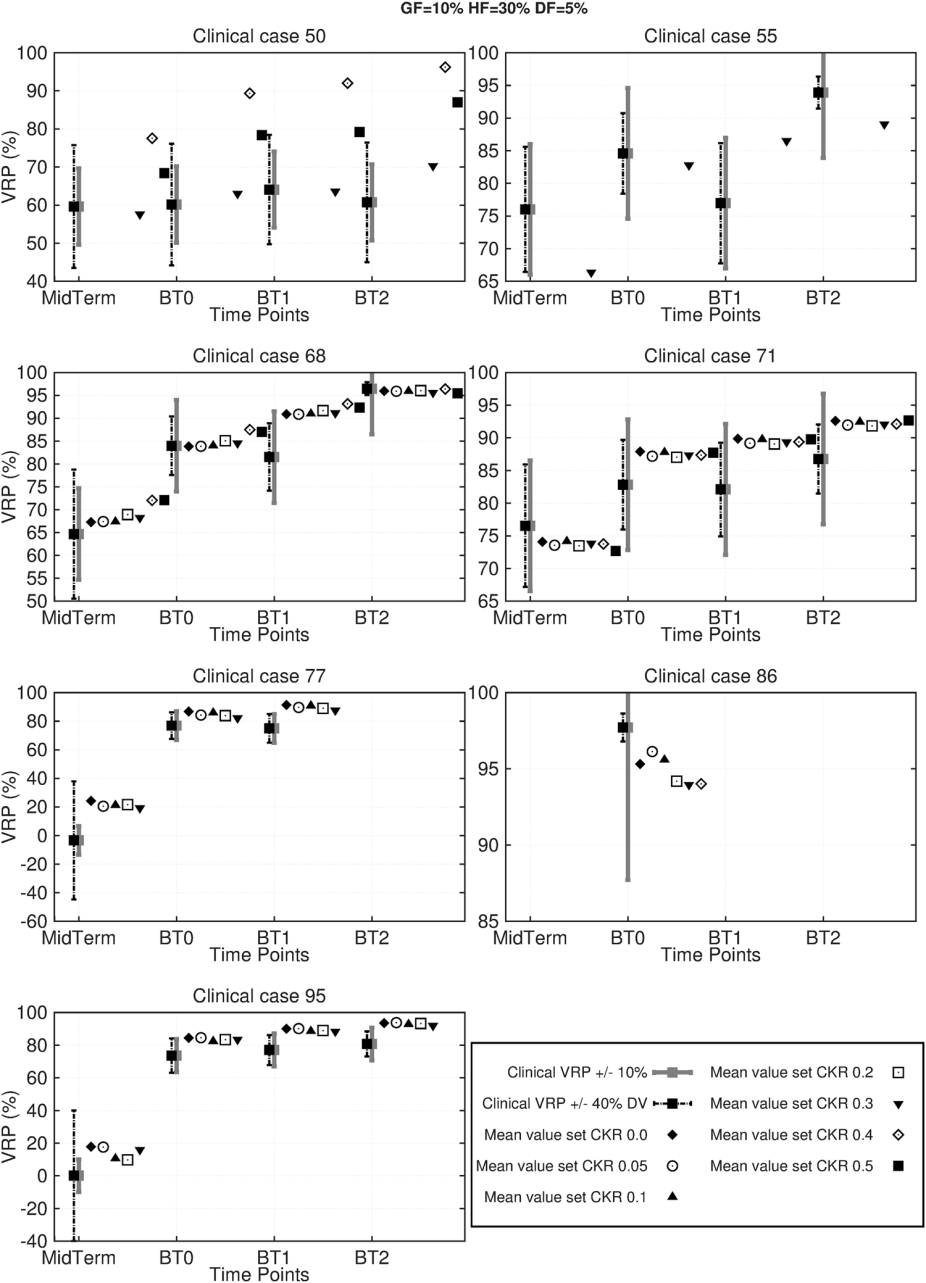
Simulations with mean value parameter sets for the low proliferative tumour profile. Comparison of clinical and simulated Volume Reduction Percentages (VRP), for the tumour profile GF = 10%, HF = 30%, DF = 5%. The simulated VRP at each time-point is compared to the corresponding clinical VRP. The error bars indicate the permitted variability around the clinical VRP value according to the Criteria VRP 10% (equation (11)) and 40% DV (equation (13)).This variability is introduced to reflect possible tumour delineation errors. VRP values are computed according to equation (9). Note: For each timepoint (midterm, BT0, BT1, BT2) the VRPs for each CKR value are horizontally displaced to facilitate the inspection of the results; this displacement does not have temporal meaning.

Subsequently, the mean value parameter sets were constructed and new simulation runs have been performed, in order to test their capability to represent each tumour profile/CKR value combination. Figures 3 and 4 present these results, by comparing clinical with simulated VRPs. Table 2 presents the corresponding MAE errors (equations (15, 16)). The use of the mean value parameter sets proves to be an efficient way to characterize a patient/tumour profile/CKR value combination. In the majority of cases the mean value parameter sets constitute themselves solutions. In most cases where a mean value parameter set is rejected based on the formal volumetric criteria, the deviations are small as indicated by their MAE values.

**Table 2:** MAE errors for the simulations performed with the mean value parameter sets.

Patient		50	55	68	71	77	86	88	95
Tumour profile: GF 60% HF 30% DF 5%	**CKR**	${\boldsymbol{MA}}{{\boldsymbol{E}}}_{{\boldsymbol{clinical}}{\boldsymbol{case}},{\boldsymbol{tumour}}{\boldsymbol{profile}},{\boldsymbol{ckr}}{\bf{value}}}$ **(**Equation (15)) **(%)**							
	**0**	—	—	2.77	—	4.85	2.22	1.70	8.12
	**0.05**	—	—	4.36	—	7.15	2.23	1.48	6.93
	**0.1**	—	—	3.88	—	7.19	2.28	2.97	5.83
	**0.2**	9.72	—	3.16	—	5.80	2.28	1.55	7.04
	**0.3**	6.50	—	2.97	—	3.03	2.28	1.54	5.49
	**0.4**	5.46	—	4.74	—	3.60	2.27	—	5.36
	**0.5**	6.68	6.86	4.55	—	—	2.23	—	5.13
		${\boldsymbol{MA}}{{\boldsymbol{E}}}_{{\boldsymbol{clinical}}{\boldsymbol{case}},{\boldsymbol{tumour}}{\bf{\text{profile}}}}$ **(**Equation(16)**) (%)**							
		**7.09**	**6.86**	**3.78**	**—**	**5.27**	**2.26**	**1.85**	**6.27**
Tumour profile: GF 10% HF 30% DF 5%	**CKR**	${\boldsymbol{MA}}{{\boldsymbol{E}}}_{{\boldsymbol{clinical}}{\boldsymbol{case}},{\boldsymbol{tumour}}{\boldsymbol{profile}},{\boldsymbol{ckr}}{\boldsymbol{value}}}\,$ **(**Equation (15)**) (%)**							
	**0**	—	—	3.25	5.25	18.00	2.39	—	13.50
	**0.05**	—	—	3.25	6.00	15.33	1.58	—	13.75
	**0.1**	—	—	3.25	5.25	16.33	2.13	—	10.50
	**0.2**	—	—	4.00	5.25	15.33	1.84	—	11.00
	**0.3**	3.75	6.50	3.75	6.00	13.67	1.91	—	12.25
	**0.4**	27.75	—	5.75	5.00	—	2.15	—	—
	**0.5**	17.00	—	5.50	5.50	—	2.07	—	—
		${\boldsymbol{MA}}{{\boldsymbol{E}}}_{{\boldsymbol{clinical}}{\boldsymbol{case}},{\boldsymbol{tumour}}{\bf{profile}}}$ **(**Equation (16)**) (%)**							
		**13.5**	**6.5**	**4**	**5.25**	**15.67**	**2.01**	—	**12.25**

A special note should be made about patient 86, for which volumetric data were available for two time points only (pre-therapy and BT0) which is equivalent to a more relaxed constraint. As expected, longitudinal model fitting in this case is generally easier compared to the cases where more time-points are available. As a result, the simulation returns a large number of accepted parameter value sets with very small MAE values. It is expected that many of these parameter value sets would be rejected if more time points with volumetric data were available. This observation stresses the importance of acquiring longitudinal volumetric and other data, and reveals the advantage of modeling approaches able to handle such longitudinal data.

In order to get tumour scenarios compatible with the clinical data, the low proliferative tumour profile retrieves in general tumour scenarios of higher radiosensitivity and larger tumour doubling times, compared to the high proliferative profile. No important differences are observed with regards to the tumour cell cisplatin chemosensitivity (Supplementary Table S22).

The constraint of volumetric data coupled with the choice of the highly proliferative profile leads to radiosensitivity values lying close to the lower end of the literature-reported range (range of mean values of the alpha parameter of the LQ model: 0.011–0.377 Gy^−1^) (Supplementary Table S21). On the other hand, the low proliferative profile leads to a wider distribution of mean radiosensitivity values (range: 0.007–0.510 Gy^−1^) (Supplementary Table S12). In addition, this low proliferative profile results in an approximate classification of the tumours with respect to their radiosensitivity that reflects their comparative regression profiles as depicted in Supplementary Fig. S1. To the tumours presenting slower clinical regression (50, 68, 77, 95) are assigned lower radiosensitivity mean values compared to the ones with steeper clinical regression “slopes” (55, 71, 86). Another interesting observation relates to the values of the cell cycle duration. The low proliferative profile results in mean T_C_ values (17–39 h) that are much closer to the mean values reported in literature compared to the high proliferative one (45–62 h).

### Results of predictive tests

**Figure 5:**
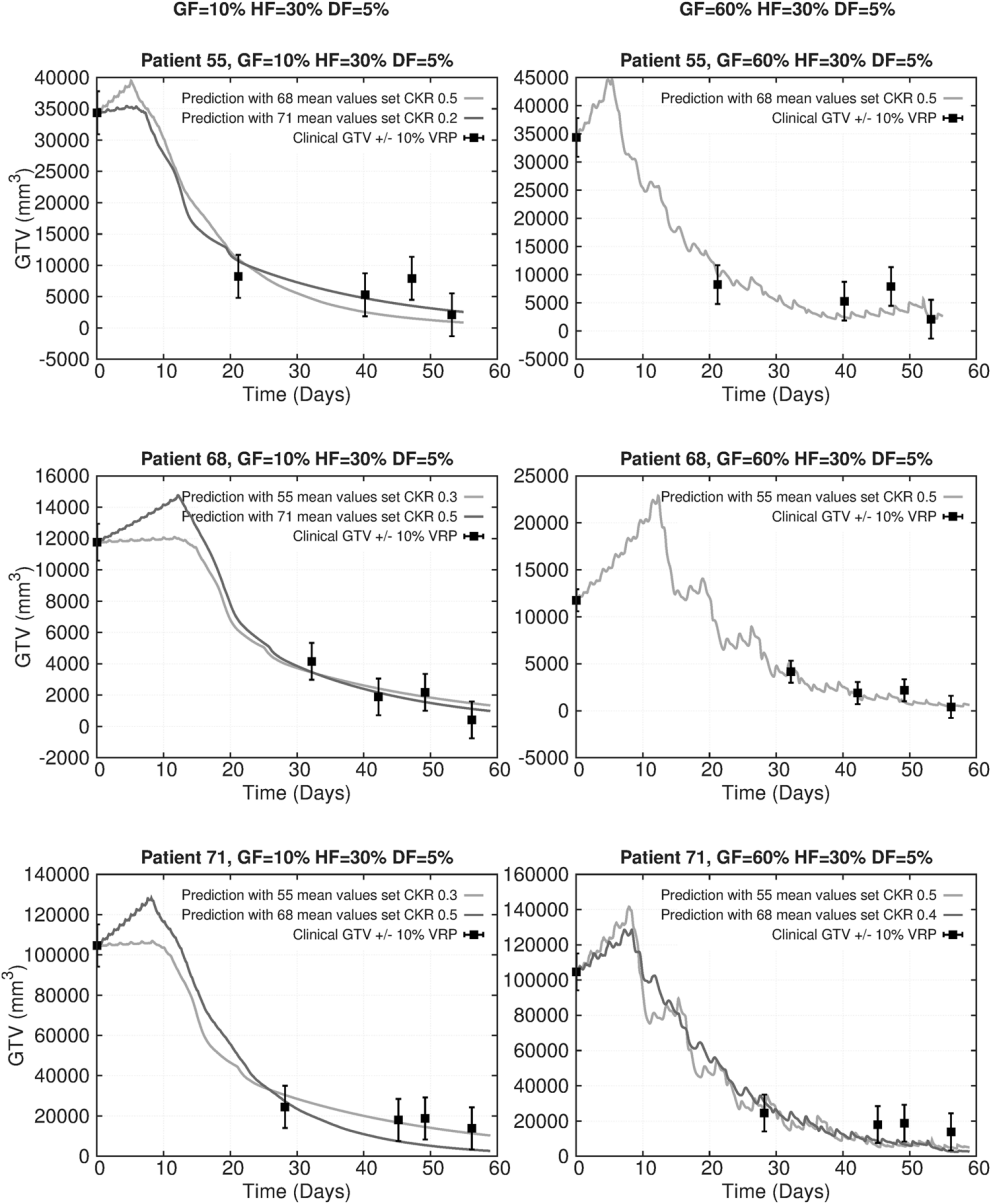
Predictive tests with patients 55, 68, 71. Left panel: GF = 10%, HF = 30%, DF = 5%. Right panel: GF = 60%, HF = 30%, DF = 5%. Tumour volume time course for patients 55 (1st row), 68 (2nd row) and 71 (3rd row) using the mean value sets of the other two patients. 0: pre-therapy timepoint. The error bars indicate the permitted variability around the clinical VRP value according to the criterion VRP 10 (equation (11)) for the time points pre-therapy, midterm, BT0, BT1, BT2 successively. This variability is introduced to reflect possible tumour delineation errors.

By using the MAE pair error method, a sorting of the all patient pairs has been derived, reflecting a quantitative estimation of their similarity and mutual predictive ability in tumour regression terms (Table 3). MAE pair errors up to about 10% correspond to very good prediction results. MAE errors in the range 0–4% correspond to the Criterion VRP 5 mostly. There are three patients (55, 68, 71) that seem to belong to a group of similar patients, for which all pairs that can be formed are characterized by relatively low MAE errors. These pairs reside in the upper row of Table 3. An alternative reading is that we can use anyone of these patients to predict the evolution of the tumours of the other two with very good results (Fig. 5). The regression profile of these three tumours seems very similar as indicated by their tumour volume reduction percentages at all considered time points (Supplementary Table S1); this has been reflected in their pair MAE errors.

**Table 3:** MAE pair errors in ascending order. A and B denote the first and the second item, respectively, of each two-patient pair.

GF 10% HF 30% DF 5%				GF 60% HF 30% DF 5%			
Pair (A-B)	${\boldsymbol{MA}}{{\boldsymbol{E}}}_{{\boldsymbol{A}}\leftrightarrow {\boldsymbol{B}}}$(%) (Equation (17))	${\boldsymbol{MA}}{{\boldsymbol{E}}}_{{\boldsymbol{A}}\leftarrow {\boldsymbol{B}}}$ (Equation (16))	${\boldsymbol{MA}}{{\boldsymbol{E}}}_{{\boldsymbol{B}}\leftarrow {\boldsymbol{A}}}$(%) (Equation (16))	Pair (A-B)	${\boldsymbol{MA}}{{\boldsymbol{E}}}_{{\boldsymbol{A}}\leftrightarrow {\boldsymbol{B}}}$(%) (Equation (17))	${\boldsymbol{MA}}{{\boldsymbol{E}}}_{{\boldsymbol{A}}\leftarrow {\boldsymbol{B}}}$(%) (Equation (16))	${\boldsymbol{MA}}{{\boldsymbol{E}}}_{{\boldsymbol{B}}\leftarrow {\boldsymbol{A}}}$(%) (Equation (16))
55–71	4.6	6.0	3.3	55–68	7.0	8.8	5.3
68–71	7.4	5.3	9.5	77–95	8.8	8.3	9.3
55–68	8.1	10.8	5.5	68–71	9.0	—	9.0
88–68	9.7	9.7	—	50–71	9.3	—	9.3
88–95	10.3	10.3	—	55–71	10.0	—	10.0
88–77	10.7	10.7	—	88–55	12.4	12.3	12.5
88–50	11.0	11.0	—	88–77	13.3	10.3	16.3
88–71	12.0	12.0	—	50–55	13.3	18.5	8.0
77–68	12.9	21.3	4.5	50–68	13.4	20.8	6.0
77–95	13.3	7.0	19.5	77–71	14.3	—	14.3
88–55	13.3	13.3	—	95–88	14.5	12.8	16.3
50–71	13.6	17.8	9.5	88–68	15.9	9.3	22.5
77–55	15.5	21.5	9.3	88–50	17.8	12.0	23.5
77–71	16.1	24.7	7.5	88–71	17.8	—	17.8
50–55	16.4	21.3	11.5	50–77	20.0	22.0	18.0
50–68	16.5	27.0	6	95–68	20.9	22.5	19.3
95–55	16.5	21.0	12.0	77–68	21.5	22.0	21.0
95–71	16.5	23.8	9.25	95–55	21.6	24.0	19.3
95–68	17.1	22.8	11.5	77–55	22.0	32.0	12.0
95–50	19.4	20.3	18.5	95–50	23.1	17.8	28.5
50–77	20.7	22.8	18.7	95–71	23.3	—	23.3
86–68	15.8	21.0	14.5	86–55	14.4	11.8	17.0
86–88	16.0	—	16.0	86–71	18.0	—	18.0
86–71	16.4	18.0	14.8	86–68	18.2	18.1	18.3
86–55	16.9	22.0	11.8	86–88	26.7	33.7	19.7
86–50	28.5	21.0	36.0	86–50	29.2	19.7	38.8
86–77	29.5	21.0	38.0	86–77	41.4	40.5	42.3
86–95	33.1	32.0	34.3	86–95	42.9	48.1	37.8

Pairs incorporating cases 50, 77, 88, and 95 are characterized by higher MAE errors. Pairs 77–95 and 50–71 exhibit low MAE errors for the highly proliferative profile only. The last seven rows of the sorting list complete the similarity picture derived by our methodology and are occupied by pairs in which patient 86 participates. These are presented separately, because, as explained previously, volumetric data for two time-points only is equivalent to a more relaxed constraint.

With the only exception of 50–71 in the highly proliferative profile, all other pairs incorporating case 50 are generally characterized by a comparatively very high MAE error. This result can be explained based on the observation that patient’s 50 regression profile is remarkably different; it displays a limited and practically constant tumour regression (with VRPs: midterm: 59.63%, BT0: 60.14%, BT1: 64.07%, BT2: 60.72%).

A similar observation holds true for the vast majority of pairs incorporating cases 77 or 95. These clinical cases are exceptional because they practically show no tumour shrinkage at the midterm timepoint (clinical VRPs of −3.37% and 0.13%, respectively) and low tumour regression subsequently. This is a highly plausible explanation for their relative inadequacy to predict or be predicted by the other clinical cases; particularly so since their mutual similarity and predictive ability is high.

The above constitute a strong indication that the pair methodology, through the use of the mean value sets, is promising in differentiating in a quantifiable way tumours with different regression profiles. The similarity of the regression profiles is reflected in the assigned MAE errors. Pairs formed by cases with similar regression profiles are assigned lower MAE errors compared to pairs formed by tumours with highly divergent regression profiles.

## Discussion

One of the main aims of this study was the design of a clinically-meaningful parameterization methodology for the CERvical cancer ONCOsimulator. This was accomplished through a tumour features-based search of the parameter space. The methodology was applied to eight cervical cancer patients.

For each patient, the simulations retrieve adaptation solutions, i.e. sets of model parameter values that result in virtual tumours whose evolution is compliant with: (a) the chosen tumour profile, (b) clinical data (e.g. longitudinal tumour volumetric data), and (c) biologically plausible value ranges of the model parameters and virtual tumour features. Each solution belongs to a specific tumour profile family of solutions and represents a distinct tumour scenario compatible with the data.

A methodological framework for the predictive use of the model was also sought. As a first step towards this direction, we have studied the solutions of each tumour profile for each patient, and new parameter value sets were created by assigning to each parameter the mean of the corresponding values in the solution family. This part of our study revealed that each profile of each patient can be adequately represented by a single parameter value set for each CKR value tested.

A series of predictive investigations was subsequently performed. These were based on the consideration of patient pairs, wherein the single parameter value set assigned to a specific patient and tumour profile is used to predict the volumetric evolution of another patient’s tumour for the same profile (and vice versa). While bearing in mind all the shortcomings resulting from the limited number of patients, the methodology permitted a quantitative estimation of the tumours’ similarity and bilateral predictive ability in terms of their regression profile. Three patients have been identified, anyone of which can be used to predict the evolution of the tumours of the other two with very good results. At the same time, clinical cases with “outlier” regression profiles proved inadequate for the same purpose, supporting the relevance of the approach. Our observations show that the presented approach is promising in differentiating, in a quantifiable way, tumours with different regression profiles, a result that supports the model’s use in a predictive setting.

The simulation results indicate the inherent capacity of CERONCO to discern tumour profiles that are compatible with the actual evolution of a clinical tumour from incompatible ones, thereby complementing the characterization of a tumour when experimental/clinical information is lacking. When many different scenarios are in accordance with the observed tumour behaviour, CERONCO can specify which clinical/experimental information could be sought to narrow down the number of compatible tumour scenarios. It can also suggest plausible value ranges for currently unidentified parameters.

Our results also show that agreement with clinical data in volumetric terms alone may mask tumours with radically different characteristics and, hence, prognosis. This can be illustrated intuitively in the form of tumour relapse experiments, where tumours of different profiles, all compliant with the longitudinal volumetric data, may have a very different constitution in terms of the distinct tumour cell subpopulations having survived the treatment. These different post-therapy tumour constitutions are expected to display variable tumour regrowth potential. Such studies with CERONCO are ongoing and planned to form the subject of a dedicated publication.

All these observations carry important implications for the future clinical validation of CERONCO. A formal validation framework can be briefly outlined as follows:

- **Parameter estimation**:

- CERONCO parameter estimation for a subset of patients (*training set*), using a dense search of the tumour profile space. The number of candidate profiles can be reduced if specific information about the tumour profile or other features is available, through imaging or other studies, as well as if follow-up data are made available.
- Identification of a single representative parameter value set for each tumour profile/patient combination of the training set (the mean value parameter set is a strong candidate; a special handling has to be devised for the transition from the profile/patient/CKR value mean sets to profile/patient ones).

- **Classifier construction:**
  Use of machine learning approaches for the identification of features that can define different patient classes. The goal will be to derive triple combinations of the form: patient class/tumour profile/assigned parameter value set.

- **Validation**:
  Use of an independent set of patients (*validation set*) to evaluate predictive simulations, based on each patient’s class (use of the parameter value set identified previously for each profile of a patient class).

Clinical validation of complex multiscale models is a demanding long-term process, presupposing extensive interdisciplinary effort to overcome numerous challenges. The presented proof-of-concept results lend support to the possibility of using CERONCO for the prediction of response of cervical tumours to the considered treatment protocol, provided that rich clinical datasets are made available. It should be noted that the core modeling algorithms and the presented methodology is fairly easily applicable to other types of cancer as well.

## Supplementary information

Supplementary Material for revised manuscript

## Data Availability

All the datasets generated during and/or analysed during the current study, apart from the imaging data, are included in the manuscript. Imaging data were used under license for the current study and are available with restrictions from Aarhus University, Denmark, upon reasonable request.
