# Supplementary material for: Studying the regression profiles of cervical tumours during radiotherapy treatment using a patient-specific multiscale model
Source: Sci Rep. 2019 Jan 31;9:1081. doi: 10.1038/s41598-018-37155-9 (PMC6355788; doi:10.1038/s41598-018-37155-9)
Supplement: Supplementary file 1 — Supplementary Material for revised manuscript [file 41598_2018_37155_MOESM1_ESM.pdf]

SUPPLEMENTARY MATERIAL

Studying the regression profiles of cervical tumours during radiotherapy treatment using a patient-specific multiscale model.

Christos A. Kyroudis[1], Dimitra D. Dionysiou[1], Eleni A. Kolokotroni[1], Georgios S. Stamatakos[1]

[1]In Silico Oncology and In Silico Medicine Group, Institute of Communication and Computer Systems, School of Electrical and Computer Engineering, National Technical University of Athens, Athens, Greece

SUPPLEMENTARY MATERIAL SECTION SA

BRACHYTHERAPY TREATMENT MODELLING

(Derivation of the equation used within CERONCO to compute the survival fraction after each successive PDR pulse).

The basis of tumour response to brachytherapy treatment modeling is the modified Linear Quadratic model with correction for incomplete repair of Pulsed Dose Rate Brachytherapy (1),(2),(3). Considering a fraction of pulsed brachytherapy consisting of N pulses of dose d and an inter-pulse interval of the order of one hour, sub-lethal damage may not completely be repaired and the final cell survival fraction is given by:

$SF_N(d)=\exp[-(\alpha Nd+\beta NG_Nd^2)]$ (S1)

where G_N, the Lea and Catcheside factor, is calculated from the temporal characteristics of the dose distribution. Equation (S1) assumes monoexponential repair kinetics of the beta component of radiation damage (see equation S6 below). Several studies addressing the issue of G Factor calculation for pulsed brachytherapy can be found in literature. The following equations have been used for the calculation (2), (4):

$$G_N(PDR) = \frac{2}{\mu t}\left[1 - \frac{NY-S}{N\mu t}^{2}\right] \qquad \text{(S2)}$$

$$Y = 1 - e^{-\mu t} \qquad \text{(S3)}$$

$$S = \frac{NK-K-NK^2e^{-\mu t}+K^{N+1}e^{-\mu Nt}}{(1-Ke^{-\mu t})^2} \qquad \text{(S4)}$$

$$K = e^{-\mu x} \qquad \text{(S5)}$$

$$\mu = \frac{ln}{T_{1/2}} \qquad \text{(S6)}$$

where:

t is the duration of each pulse,

x is the time between pulses without irradiation,

μ is the repair rate constant,

and $T_{1/2}$ is the half time for sub-lethal damage repair. Monoexponential recovery kinetics is assumed.

In order to simulate within CERONCO, where a time step of one hour is used, the number of cells that are lethally hit by each successive pulse the following consideration has been made:

Let P_{init} be the initial tumour cell population. According to the modified LQ model, after N pulses of dose d have been applied the remaining (survived) tumour cell population will be:

$$P_N = P_{init}\, SF_N = P_{init}\, \exp(-\alpha N d - \beta N G_N d^2) \quad \text{(S7)}$$

The number of surviving tumour cells just after each successive pulse in the N-pulse scheme is given by:

$$P_1 = P_{init} \exp(-\alpha d - \beta G_1 d^2)$$

$$P_2 = P_{init}\, \exp(-\alpha 2d - 2\beta G_2 d^2)$$

$$P_3 = P_{init}\, \exp(-\alpha 3d - 3\beta G_3 d^2)$$

…

$$P_{N-2} = P_{init}\, \exp(-\alpha(N-2)d - (N-2)\beta G_{N-2} d^2)$$

$$P_{N-1} = P_{init}\, \exp(-\alpha(N-1)d - (N-1)\beta G_{N-1} d^2)$$

$$P_N = P_{init}\, \exp(-\alpha N d - \beta N G_N d^2)$$

Since each pulse acts upon the tumour cell population that remained alive after all previous pulses the following equation applies for every two successive pulses:

$$P_i = P_{i-1} SF_i \quad , \text{i} = 1,\ldots, \text{N (S8)}$$

Where SF_i is the survival fraction after the i-th pulse of the N-pulse scheme.

The goal is to derive a suitable mathematical formulation of SF_i, so that the correct number of lethally hit cells is computed within CERONCO at the appropriate time points, just after each pulse dose.

(S7), (S8) =>

$$P_{init}\, \exp(-\alpha i d - \beta i G_i d^2) = P_{init}\, \exp(-\alpha(i-1)d - \beta(i-1)G_{i-1} d^2)\, SF_i$$

$$\Rightarrow \; SF_i = \frac{\exp(-\alpha i d - \beta i G_i d^2)}{\exp[-\alpha(i-1)d - \beta(i-1)G_{i-1} d^2]}$$

$$\Rightarrow \; SF_i = \exp(-ad)\exp[-\beta d^2(iG_i - (i-1)G_{i-1})], \; \text{i=1…N} \quad \text{(S9)}$$

Therefore, within CERONCO equation (S9) will be applied after each successive pulse.

G_i is computed by Eq. (S2)-(S6) for N=i, $\forall\, i \geq 2$,

and

$$G_1 = \frac{2\,[\exp(-\mu t) + \mu t - 1]}{(\mu t)^2} \quad \text{(S10) for the first pulse}$$

(this is the appropriate equation for one dose of radiation delivered at constant dose rate during time interval t (5)). Eq. (S10) can be derived from Eq. (S2)-(S6) for N=1 and x->∞.

Eq. (S9) is in essence a modification of the LQ model that can be used within CERONCO after each successive pulse and takes into account the current number of living tumour cells. This number is defined by the competing processes of cell death (due to radiotherapy, apoptosis, necrosis) and cell birth; these processes are incorporated in the cytokinetic model of Fig 1 of the main text.

In the pulsed brachytherapy scheme considered in the context of this study two brachytherapy fractions of 20 pulses each are used. The pulse dose is derived for each geometrical cell of the tumour mesh from the corresponding total dose distribution raw file by dividing the total dose corresponding to the specific geometrical cell by the number of pulses. The interval between successive pulses is 1 hour and the pulse duration varies among clinical cases (e.g. approximately 0.2-0.3 hours, i.e. time between pulses without irradiation: 0.8-0.7h).

SUPPLEMENTARY MATERIAL SECTION SB:

SB.1: THE CLINICAL DATA

Eight clinical cases of cervical cancer have been included in the study. The patients were treated as part of the large prospective multicenter EMBRACE clinical study (6). Supplementary Table S1 presents a summary of the available clinical data. Follow-up data were not available. 01 provides a schematic description of the evolution of the tumours' Gross Tumour Volume (GTV) throughout therapy.

Supplementary Table S1. Summarized description of basic features of the available patient cases.
SCC: *Squamous cell carcinoma.*
GTV: *MRI-derived Gross Tumour Volume*
EBRT: *External Beam Radiotherapy*
Pretherapy: *Before start of treatment.*
Day 0: *pretherapy GTV based on MRI*
Midterm: *During EBRT*
BT0: *Before start of brachytherapy treatment*
BT1: *Start of 1st brachytherapy fraction*
BT2: *Start of 2nd brachytherapy fraction*
NA: *Not Available.*
Initial equivalent diameter: the diameter of a sphere with the same volume as the actual initial tumour.

VRP: *Volume Reduction Percentage,* $\boldsymbol{VRP} = \left({(\boldsymbol{GTV_{Pretherapy}} - \boldsymbol{GTV_{Timepoint}})} \middle/ {\boldsymbol{GTV_{Timepoint}}} \right) * \mathbf{100\%}$,

Timepoint $\in$ *{Midterm, BT0, BT1, BT2}*

	**Clinical case**							
	**50**	**55**	**68**	**71**	**77**	**86**	**88**	**95**
**Histology**	SCC	SCC	SCC	SCC	SCC	SCC	SCC	SCC
**FIGO stage**	4A	2B	2B	3B	2B	2B	2B	2B
**Differentiation degree**	NA	low	NA	low	NA	NA	NA	low
**Untreated time period (before EBRT start) (days)[a]**	8	4	11	7	3	7	1	2
**EBRT Total dose (Gy)/no of fractions Duration[a]**	45/25 Day 9 - Day 43	45/25 Day 5 - Day 41	50/30 Day 12 - Day 54	50/30 Day 8 - Day 54	45/25 Day 4 - Day 38	45/25 Day 8 – Day 42	45/25 Day 2- Day 38	50/30 Day 3- Day 31

**No of cisplatin chemo cycles**	3 (2 lost)	5	6	6	5	5	5	5
**Chemotherapy schedule**	Weekly cisplatin 40mg/m^2 (1st administration: start of EBRT)							
**PRETHERAPY MRI-derived GTV (mm^3): (Equivalent diameter) (mm)**	22790 (35.18)	34352 (40.34)	11767 (28.22)	104633 (58.46)	27626 (37.5)	44131 (43.84)	46148 (46.15)	19466 (33.38)
**Necrosis diameter at pretherapy (mm)**	no	no	no	yes (18mm)	no	no	no	no
**MIDTERM MRI-derived GTV (mm^3) [VRP (%)]**	9201 [59.63]	8238 [76.02]	4157 [64.67]	24532 [76.55]	28556 [-3.37]	NA	21037 [54.41]	19440 [0.13]
**BT0 MRI-derived GTV (mm^3) [VRP (%)]**	9083 [60.14]	5300 [84.57]	1886 [83.97]	17968 [82.83]	6370 [76.94]	1009 [97.71]	2366 [94.87]	5138 [73.61]
**BT1 MRI-derived GTV (mm^3) [VRP (%)]**	8188 [64.07]	7913 [76.96]	2174 [81.52]	18719 [82.11]	6920 [74.95]	NA	3770 [91.83]	4463 [77.07]
**BT2 MRI-derived GTV (mm^3): [VRP (%)]**	8951 [60.72]	2097 [93.90]	415 [96.47]	13846 [86.77]	NA	NA	NA	3751 [80.73]
**Midterm[a]**	Day 32	Day 22	Day 33	Day 29	Day 15	NA	Day 27	Day 15
**BT0[a]**	Day 44	Day 41	Day 43	Day 46	Day 37	Day 36	Day 37	Day 42
**BT1[a]**	Day 51 (20 pulses)	Day 48 (20 pulses)	Day 50 (20 pulses)	Day 50 (15 pulses)	Day 44 (20 pulses)	NA	Day 42 (20 pulses)	Day 49 (20 pulses)
**BT2[a]**	Day 58 (20 pulses)	Day 54 (20 pulses)	Day 57 (20 pulses)	Day 57 (20 pulses)	NA	NA	NA	Day 56 (20 pulses)

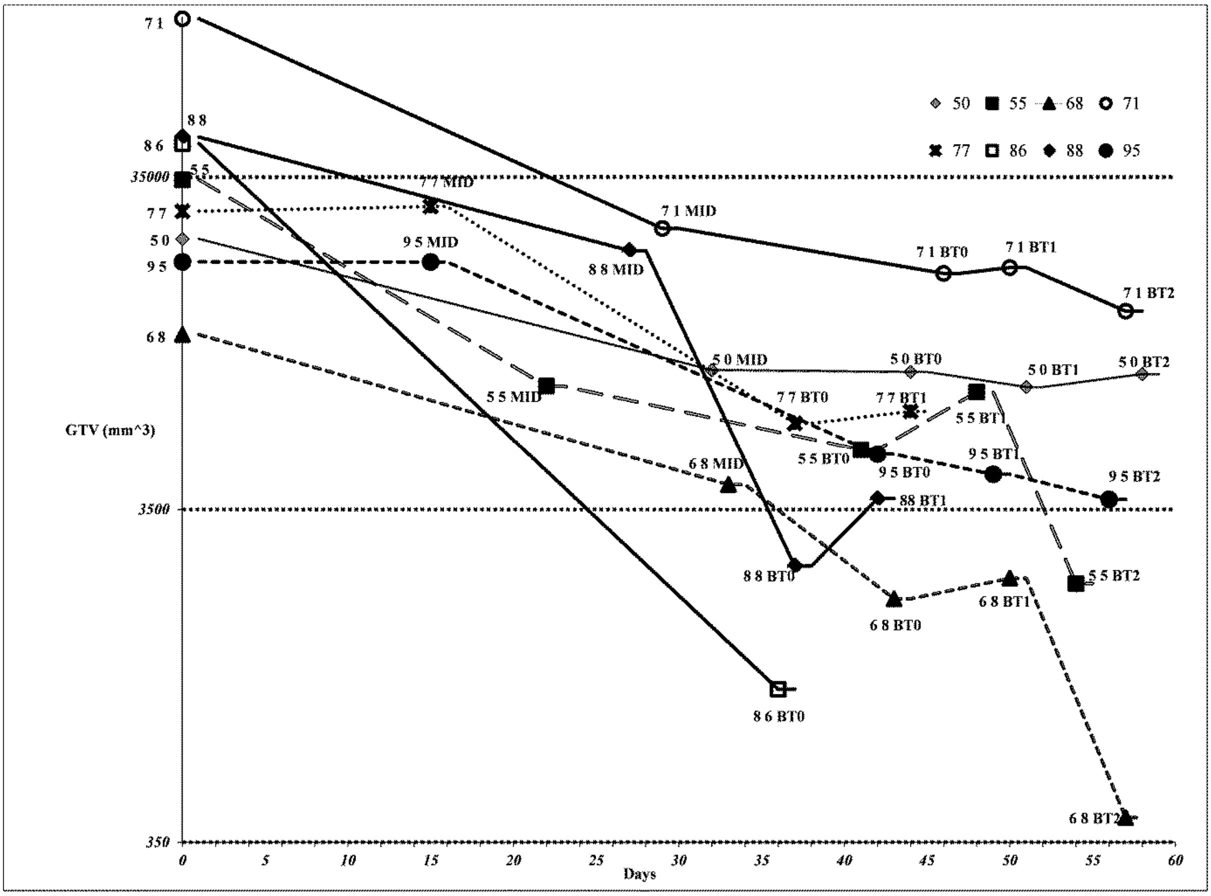

Supplementary Figure S1. Semilogarithmic plot of the Gross Tumour Volume (GTV) of the clinical cases at several timepoints. *Available timepoints: Pretherapy, Midterm (MID)(during EBRT), BT0 (Before BT start), BT1 (start of 1st BT fraction), BT2 (start of 2nd BT fraction). Day 0 corresponds to the day of acquisition of the pretherapy GTV. For the temporal features of each patients treatment schedule see Supplementary Table S1. Note: the lines connecting the GTV data points are provided to facilitate the understanding of the rate of tumour shrinkage in each case; they do not imply that tumour evolution over time in between the available GTV data points follows this linear pattern.*

SB.2: IMAGING DATA PREPROCESSING

Algorithms and code have been developed in order to transform the imaging data into a form appropriate to be used as an input to CERONCO. For each clinical case the following files are created as an input for CERONCO:

1) 3D-reconstructions of the Gross Tumour Volume (GTV) (raw files, tumour or non/tumour per GC) for (up to) 5 time points: Pretherapy (before start of treatment), Midterm (during external beam radiotherapy treatment), BT0 (before brachytherapy treatment), BT1 (start of 1st brachytherapy fraction), BT2 (start of 2nd brachytherapy fraction)
2) BT1, BT2 spatial dose distribution raw files (dose per GC).

These 3D-reconstruction raw files supply the model with the tumour's spatial information and correspond to the region of interest onto which the discretizing mesh of the model is superimposed. Within each raw file each Geometrical Cell (GC) of the mesh is labeled as tumour or non/tumour. The procedure for the creation of these input files is the following:

- A Reference CT is acquired prior to therapy
- A Reference GTV is derived from the reference CT
- The MRI images of all available time points (pretherapy, mid-term, BT0, BT1, BT2) are registered to the reference CT.
- GTV files are derived from each MRI
- The MRI GTV files are registered to the Reference GTV (registration to the Reference GTV center of mass)
- The same procedure is used to produce the raw files for the BT doses

T2 weighted MRI with 4mm slice thickness was acquired according to the GEC ESTRO guidelines (7).

SUPPLEMENTARY SECTION SC

THE TUMOUR PROFILE CONCEPT

Supplementary Table S22 provides the mathematical relationships between tumour features (e.g. Growth Fraction, GF, Hypoxic Fraction, HF, etc.) and CERONCO parameter values (8). The computation of the various initial tumour cell populations is also explained. There is a number of independent CERONCO parameters, randomly taking values within predefined ranges according to literature; these are: T_C, T_A, OER, R_A, R_{ADiff}, P_{G0toG1}, $P_{sym,}$ α, stem_cell_percentage. The dependent parameters of the multi-constraint adaptation algorithm are the following: β, P_{sleep}, R_{NDiff}, $T_{G0,}$ T_N, N_{LIMP}; the latter are computed based on the assigned values of the independent parameters and the user-selected features of the tumour (see main text of the article: The tumour profile concept). NA: non-applicable.

Supplementary Table S2. ***Computation of CERONCO parameter values based on the chosen tumour profile (GF, HF, DF) and the chosen value of the tumour's doubling time (T_d).*** *For the description of the CERONCO parameters see Table 1 of the main article.*

**Model parameter or virtual tumour feature**	**Description**	**Computation**
**Total**	Initial total number of tumour cells	Computed based on the imaging-based GTV tumour volume, by considering a typical solid tumour cell density (e.g. 10^9 cells/cm^3), unless more specific information for a particular tumour is available.
**DF (%)**	Initial tumour dead fraction (Percentage of dead tumour cells over all tumour cells).	**Defined by the user.** In the current study its estimation was based on the provided initial tumour's necrotic diameter.
**Dead**	Initial number of dead tumour cells.	Dead = DF*Total
**Living**	Initial number of living tumour cells.	Living= Total –Dead.
**HF (%)**	Hypoxic fraction. Percentage of hypoxic (dormant)	**Defined by the user.**

	tumour cells over all tumour cells.	
**Hypoxic**	Initial number of hypoxic (dormant) tumour cells.	Hypoxic = HF*Total
**GF (%)**	Growth fraction. Percentage of proliferating tumour cells over all living tumour cells.	**Defined by the user.**
**Prolif**	Initial number of proliferating tumour cells.	Prolif = GF*Living
**DIFF**	Initial number of terminally differentiated tumour cells.	DIFF =Living-Prolif-Hypoxic
**Stem_Cell_Percentage (%)**	Initial percentage of stem cells over all living tumour cells.	Random value ∈ [0.01-0.12]
**N_S**	Initial number of stem cells.	N_S = Stem_Cell_Percentage * Living
**N_L**	Initial number of LIMP cells.	N_L = Living-DIFF-N_S
**T_d (days)**	Initial tumour volume doubling time.	**Defined by the user**
**r (h^{-1})**	Initial tumour growth rate.	r = ln(2)/T_d/24
**CKR cisplatin**	Cisplatin Cell Kill Rate Fraction of stem and LIMP cells lethally hit at each drug administration.	**Defined by the user** ∈ {0.0, 0.05, 0.1, 0.2, 0.3, 0.4, 0.5}
**T_c (h)**	Cell cycle duration	Random value ∈ [16- 70]
**T_A (h)**	Apoptosis duration	Random value ∈ [1- 25]
**α (Gy^{-1})**	Parameter of the LQ model	Random value ∈ [0- 0.7]
**β (Gy^{-2})**	Parameter of the LQ model	Based on α value, so that α/ β=10Gy
**OER**	Oxygen enhancement ratio	Random value ∈ {1.5, 2.0, 2.5, 3.0}
**R_A (h^{-1})**	Spontaneous apoptosis rate of stem and	Random value ∈ [0.001- 0.1], step 0.001

	LIMP cells (fraction of stem and LIMP cells dying through spontaneous apoptosis per hour)	
**R_{ADiff} (h^{-1})**	Spontaneous apoptosis rate of differentiated cells (fraction of differentiated cells dying through spontaneous apoptosis per hour)	Random value ∈ [0.001- 0.1], step 0.001
**P_{G0toG1} (h^{-1})**	Fraction of dormant cells that re-enter cell cycle per hour	Random value ∈ [0.01- 0.06], step 0.01
**P_{sym}**	Fraction of stem cells at mitosis that perform symmetric division	Random value ∈ [0.20- 0.66], step 0.02
**$T_{1/2}$ (h)**	Sublethal damage repair half-time	Fixed value =1.5
**T_{G0} (h)**	G0 duration	$T_{G0} = \left[\frac{(1+P_{sym}-e^{(r+R_A)*T_C})*(r+R_A)}{(e^{(r+R_A)*T_C}-1)*\frac{HF}{GF}\left(\frac{1}{1-DF}\right)}-(r+R_A)\right]^{-1}*(1-P_{G0toG1})$
R_{NDiff} (h^{-1})	Necrosis rate of differentiated cells (fraction of differentiated cells dying through necrosis per hour)	$R_{NDiff} = \frac{(1-P_{sym})*(r+R_A)}{(e^{(r+R_A)*T_C}-1)*\frac{1}{GF}\left(1-\frac{1}{GF}-\frac{HF}{(1-DF)}\right)}-r-R_{ADiff}$
**P_{sleep}**	Fraction of stem and LIMP cells entering G0 phase after mitosis	$P_{sleep} = \frac{1-\frac{e^{(r+R_A)*T_C}}{1+P_{sym}}}{1-\frac{\frac{P_{G0toG1}}{T_{G0}}}{r+R_A+\frac{1}{T_{G0}}}}$

**T_N (h)**	Necrosis duration	$T_N = \left[\left(\frac{\frac{1-P_{G0toG1}}{T_{G0}} * \frac{HF}{GF(1-DF)} + \frac{R_{NDiff}}{GF} * \left(1 - \frac{1}{GF} - \frac{HF}{(1-DF)}\right)}{\frac{Necrotic/Living}{GF}}\right) - r\right]^{-1}$<br><br>Where:<br>$\frac{Necrotic}{Living} = \frac{DF}{1-DF} - \frac{R_A}{r+\frac{1}{T_A}} * GF + \frac{R_A}{r+\frac{1}{T_A}} * \frac{HF}{1-DF} + \frac{R_{ADiff}}{r+\frac{1}{T_A}} * \left(1 - GF - \frac{HF}{1-DF}\right)$<br>Necrotic: Initial number of necrotic tumour cells.<br>Apoptotic: Initial number of apoptotic tumour cells.<br>Necrotic + Apoptotic = Dead
**N_{LIMP}**	Number of LIMP (Limited Mitotic Potential) cell mitoses before terminal differentiation	Computed so that:<br>$\sum_{n=0}^{N_{LIMP}-1} \frac{2^n}{(1+P_{sym})^{n+1}} = \frac{1}{(1-P_{sym}) * \frac{N_s}{N_L}}$

SUPPLEMENTARY SECTION SD

RESULTS

Supplementary Table S3. *Number of solutions (model parameter value sets that result in agreement between the virtual and the clinical tumours) identified for each patient case and tumour profile studied. GF: Growth Fraction. HF: Hypoxic Fraction. DF: Dead cell fraction. CKR: cisplatin cell kill rate. For the definition of the volumetric criteria see main text of the article. In this table solutions "VRP10" correspond only to those solutions that do not belong to VRP5 or MIXED as well (i.e.* $5\% < |VRP_{simulated} - \mathrm{VRP}_{\mathrm{clinical}}| \leq 10\%$*). For patient 71 and tumour profile GF 60%, HF 30%, DF 5%, as well as for patient 88 and the tumour profile GF 10%, HF 30%, DF 5%, the simulations do not retrieve any solutions under the volumetric criteria studied. These tumours seem to be incompatible with these specific tumour profiles.*

**TUMOUR PROFILE GF=60% HF=30% DF=5%**								
**Patient**	**Criterion**	**CKR 0.0**	**CKR 0.05**	**CKR 0.1**	**CKR 0.2**	**CKR 0.3**	**CKR 0.4**	**CKR 0.5**
**50**	**VRP 5**	-	-	-	-	-	-	-
	**VRP 10**	-	-	-	-	7	5	-
	**MIXED**	-	-	-	-	11	34	-
	**40% DV**	-	-	-	14	110	98	10
	**TOTAL (289)**	-	-	-	14	128	137	10
**55**	**VRP 5**	-	-	-	-	-	-	-
	**VRP 10**	-	-	-	-	-	-	4
	**MIXED**	-	-	-	-	-	-	3
	**40% DV**	-	-	-	-	-	-	-
	**TOTAL (7)**	-	-	-	-	-	-	7
**68**	**VRP 5**	120	76	84	72	29	7	-
	**VRP 10**	256	157	143	127	71	12	-
	**MIXED**	909	716	747	808	726	274	19
	**40% DV**	-	-	-	-	-	-	-
	**TOTAL (5353)**	1285	949	974	1007	826	293	19
**71**	**VRP 5**	-	-	-	-	-	-	-
	**VRP 10**	-	-	-	-	-	-	-
	**MIXED**	-	-	-	-	-	-	-
	**40% DV**	-	-	-	-	-	-	-
	**TOTAL (0)**	-	-	-	-	-	-	-
**77**	**VRP 5**	14	17	15	14	22	5	-

	**VRP 10**	73	51	43	15	15	6	-
	**MIXED**	56	37	35	27	27	3	-
	**40% DV**	4	5	7	2	1	2	-
	**TOTAL (496)**	147	110	100	58	65	16	-
**86**	**VRP 5**	1715	1747	1777	1851	1656	1101	675
	**VRP 10**	64	71	36	48	22	9	4
	**MIXED**	-	-	-	-	-	-	-
	**40% DV**	-	-	-	-	-	-	-
	**TOTAL (10775)**	1779	1818	1813	1898	1678	1110	679
**88**	**VRP 5**	16	18	16	23	13	-	-
	**VRP 10**	2	2	-	-	-	-	-
	**MIXED**	51	41	30	29	33	-	-
	**40% DV**	-	-	1	-	-	-	-
	**TOTAL (275)**	69	61	47	52	46	-	-
**95**	**VRP 5**	-	-	-	-	-	1	2
	**VRP 10**	22	19	18	10	6	4	4
	**MIXED**	5	5	7	15	8	18	1
	**40% DV**	4	1	1	1	-	-	1
	**TOTAL (153)**	31	25	26	26	14	23	8
**TUMOUR PROFILE GF=10% HF=30% DF=5%**								
**Patient**	**Criterion**	**CKR 0.0**	**CKR 0.05**	**CKR 0.1**	**CKR 0.2**	**CKR 0.3**	**CKR 0.4**	**CKR 0.5**
**50**	**VRP 5**	-	-	-	-	40	5	2
	**VRP 10**	-	-	-	-	42	2	4
	**MIXED**	-	-	-	-	59	9	6
	**40% DV**	-	-	-	-	219	15	5
	**TOTAL (408)**	-	-	-	-	360	31	17
**55**	**VRP 5**	-	-	-	-	-	-	-
	**VRP 10**	-	-	-	-	5	-	-
	**MIXED**	-	-	-	-	-	-	-
	**40% DV**	-	-	-	-	-	-	-
	**TOTAL (5)**	-	-	-	-	5	-	-

**68**	**VRP 5**	-	-	1	-	-	-	-
	**VRP 10**	15	11	37	34	46	18	7
	**MIXED**	1133	1083	1136	1549	1490	320	116
	**40% DV**	-	-	-	-	-	-	-
	**TOTAL (6996)**	1148	1094	1174	1583	1536	338	123
**71**	**VRP 5**	-	-	1	-	3	1	-
	**VRP 10**	19	15	17	226	360	352	102
	**MIXED**	5	5	-	42	81	70	8
	**40% DV**	-	-	-	-	-	-	-
	**TOTAL (1307)**	24	20	18	268	444	423	110
**77**	**VRP 5**	-	-	-	-	-	-	-
	**VRP 10**	37	23	21	13	9	-	-
	**MIXED**	20	15	12	7	-	-	-
	**40% DV**	83	47	34	19	11	-	-
	**TOTAL (351)**	140	85	67	39	20	-	-
**86**	**VRP 5**	24	23	28	969	758	554	405
	**VRP 10**	-	-	-	478	396	288	198
	**MIXED**	-	-	-	-	-	-	-
	**40% DV**	-	-	-	-	-	-	-
	**TOTAL (4121)**	24	23	28	1447	1154	842	603
**88**	**VRP 5**	-	-	-	-	-	-	-
	**VRP 10**	-	-	-	-	-	-	-
	**MIXED**	-	-	-	-	-	-	-
	**40% DV**	-	-	-	-	-	-	-
	**TOTAL (0)**	-	-	-	-	-	-	-
**95**	**VRP 5**	6	10	9	8	6	-	-
	**VRP 10**	55	40	49	31	12	-	-
	**MIXED**	46	51	30	15	-	-	-
	**40% DV**	58	44	22	14	8	-	-
	**TOTAL (514)**	165	145	110	68	26	-	-

For patient 71 and tumour profile GF 60%, HF 30%, DF 5%, as well as for patient 88 and the tumour profile GF 10%, HF 30%, DF 5%, the simulations do not retrieve any solutions under the volumetric criteria studied. These tumours seem to be incompatible with these specific tumour profiles.

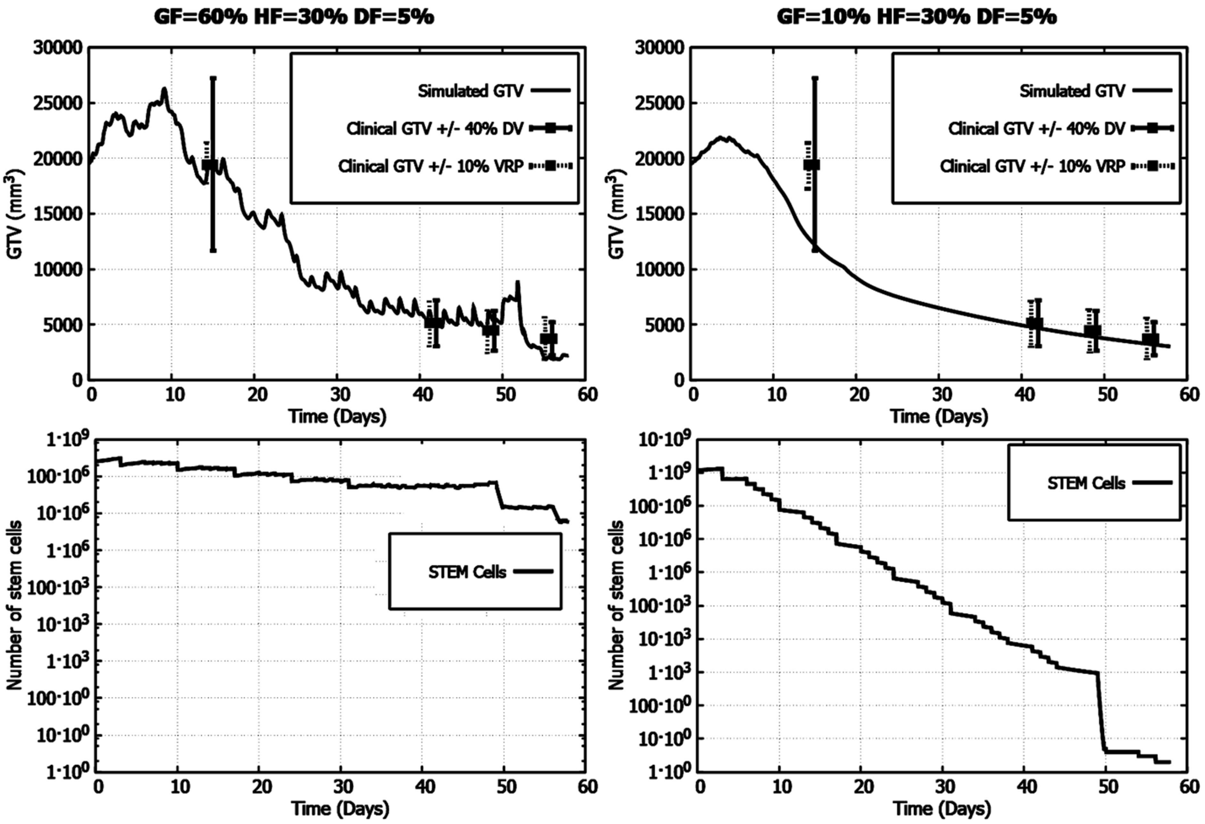

Supplementary Figure S2. *Comparison of two different tumour scenarios, both compatible with the tumour volumetric data of patient 9, in terms of GTV and number of stem cells time course. Left panel: high proliferative profile. Right panel: low proliferative profile. The same value for the tumour cells' chemosensitivity to cisplatin was chosen for both cases (CKR=0.3). The error bars indicate the permitted variability around the clinical VRP value according to the criteria VRP 10 (Eq. 11) and 40% DV (Eq. 13).*

Supplementary Table S4. *Tumour profile GF=10%, HF=30%, DF=5%, CKR: 0.0-0.5. Range of CERONCO parameter values from all identified solutions. For the definition of CERONCO parameters see Table1 of the main article. For the computation of CERONCO parameter values based on the user-defined tumour features see Supplementary Table S2. The β parameter of the LQ model is chosen so that α/β=10Gy in all simulations. The sublethal damage repair half-time $T_{1/2}$ is taken equal to 1.5Gy. T_d values represent the volume doubling time(s) of the virtual tumour(s) resulting from the mean value parameter set(s).*

**GF 10% HF 30% DF 5%, CKR 0-0.5**<br>**Range of CERONCO parameter values from all solutions**								
**Patient**	**50**	**55**	**68**	**71**	**77**	**86**	**88**	**95**
**No. of solutions**	**408**	**5**	**6997**	**1307**	**351**	**4121**	**0**	**514**
**T_d (days)**	**20-4000**	**350-1000**	**20-4000**	**20-4000**	**20-200**	**20-4000**		**20-2500**
**T_c (h)**	16-28	39-41	16-48	16-50	16-50	16-36	-	16-53
**T_{G0}(h)**	88-439	246-290	81-439	97-439	119-435	81-439	-	108-439
**T_N (h)**	6-47	19-32	6-67	6-34	6-65	6-62	-	6-74
**T_A (h)**	1-25	11-25	1-25	1-25	1-25	1-25	-	1-25
**N_{LIMP}**	3-16	10-11	3-18	4-18	3-18	3-18	-	3-18
**α (Gy^{-1})**	0.000-0.567	0.367-0.567	0.000-0.678	0.056-0.697	0.035-0.685	0.050-0.699	-	0.025-0.433
**OER**	1.5-3	1.5-3	1.5-3	1.5-3	1.5-3	1.5-3	-	1.5-3
**R_A (h^{-1})**	0.001-0.006	0.001	0.001-0.007	0.001-0.006	0.001-0.004	0.001-0.007	-	0.001-0.005
**R_{NDiff} (h^{-1})**	(06.7E-06)-0.0068	0.0004-0.0005	(3.9E-08)-0.0074	(5.4E-09)-0.002	(4.2E-07)-0.0047	(6.8E-07)-0.0075	-	(4.1E-06)-0.0058
**R_{ADiff} (h^{-1})**	0.001-0.007	0.001	0.001-0.008	0.001-0.002	0.001-0.005	0.001-0.008	-	0.001-0.005
**P_{G0toG1} (h^{-1})**	0.01-0.06	0.02-0.05	0.01-0.06	0.01-0.06	0.01-0.06	0.01-0.06	-	0.01-0.06
**P_{sleep}**	0.165-0.394	0.369-0.385	0.153-0.410	0.266-0.402	0.192-0.390	0.153-0.410	-	0.182-0.384
**P_{sym}**	0.22-0.64	0.62-0.66	0.2-0.66	0.44-0.66	0.28-0.66	0.2-0.66	-	0.26-0.66

Supplementary Table S5. *Tumour profile GF=10%, HF=30%, DF=5%, CKR=0.0. Mean value and range of values of each CERONCO parameter. For the definition of CERONCO parameters see Table1 of main article. For the computation of CERONCO parameter values based on the user-defined tumour features see Supplementary Table S2. The β parameter of the LQ model is chosen so that α/β=10Gy in all simulations. The sublethal damage repair half-time* $T_{1/2}$ *is taken equal to 1.5Gy.* T_d *values displayed as: value resulting from the mean value parameter set (range of* T_d *in all solutions).*

GF 10% HF 30% DF 5%, CKR=0.0 (For each parameter: mean value (range))								
**Patient**	**50**	**55**	**68**	**71**	**77**	**86**	**88**	**95**
**No. of solutions**	**0**	**0**	**1148**	**24**	**140**	**24**	**0**	**165**
**T_d (days)**	**-**	**-**	**127 (20-4000)**	**176 (30-3000)**	**36 (20-200)**	**105 (20-4000)**	**-**	**44 (20-2500)**
**T_c (h)**	-	-	22 (16-44)	34 (21-45)	26 (16-50)	21 (16-35)	-	27 (16-53)
**T_{G0} (h)**	-	-	253 (81-438)	285 (174-406)	302 (119-432)	266 (92-424)	-	298 (108-438)
**T_N (h)**	-	-	16 (6-62)	14 (6-22)	25 (6-59)	15 (6-30)	-	25 (6-59)
**T_A (h)**	-	-	10 (1-25)	9 (1-24)	12 (1-25)	11 (2-25)	-	12 (1-25)
**N_{LIMP} (no.)**	-	-	8 (3-18)	11 (6-18)	9 (3-18)	7 (3-10)	-	9 (3-17)
**α (Gy^{-1})**	-	-	0.084 (0.052-0.232)	0.362 (0.184-0.605)	0.204 (0.051-0.685)	0.388 (0.104-0.695)	-	0.109 (0.048-0.390)
**OER**	-	-	2.23 (1.5-3)	2.23 (1.5-3)	2.33 (1.5-3)	2.21 (1.5-3)	-	2.27 (1.5-3)
**R_A (h^{-1})**	-	-	0.0019 (0.001-0.007)	0.0013 (0.001-0.003)	0.0015 (0.001-0.004)	0.0018 (0.001-0.005)	-	0.0016 (0.001-0.005)
**R_{NDiff} (h^{-1})**	-	-	0.0019 ((2.8E-06)-0.0070)	0.00069 ((1.2E-05)-0.0012)	0.0099 ((1E-05)-0.0047)	0.0020 (0.00027-0.0043)	-	0.001 ((4.05E-06)-0.0058)
**R_{ADiff} (h^{-1})**	-	-	0.0021 (0.001-0.007)	0.0012 (0.001-0.002)	0.0015 (0.001-0.005)	0.0023 (0.001-0.005)	-	0.0014 (0.001-0.004)
**P_{G0toG1} (h^{-1})**	-	-	0.035 (0.01-0.06)	0.035 (0.01-0.06)	0.038 (0.01-0.06)	0.038 (0.01-0.06)	-	0.038 (0.01-0.06)
**P_{sleep}**	-	-	0.301 (0.154-0.410)	0.345 (0.285-0.395)	0.314 (0.192-0.389)	0.288 (0.194-0.386)	-	0.318 (0.183-0.384)
**P_{sym}**	-	-	0.485 (0.2-0.66)	0.584 (0.46-0.66)	0.534 (0.28-0.66)	0.451 (0.26-0.62)	-	0.542 (0.26-0.66)

Supplementary Table S6. *Tumour profile GF=10%, HF=30%, DF=5%, CKR=0.05. Mean value and range of values of each CERONCO parameter. For the definition of CERONCO parameters see Table1 of main article. For the computation of CERONCO parameter values based on the user-defined tumour features see Supplementary Table S2. The β parameter of the LQ model is chosen so that α/β=10Gy in all simulations. The sublethal damage repair half-time $T_{1/2}$ is taken equal to 1.5Gy. T_d values displayed as: value resulting from the mean value parameter set (range of T_d in all solutions).*

GF 10% HF 30% DF 5%, CKR=0.05<br>(For each parameter: mean value (range))								
**Patient**	**50**	**55**	**68**	**71**	**77**	**86**	**88**	**95**
**No. of solutions**	**0**	**0**	**1094**	**20**	**85**	**23**	**0**	**145**
**T_d (days)**	-	-	127 (20-4000)	301 (20-4000)	29 (20-90)	81 (30-4000)	-	30 (20-400)
**T_c (h)**	-	-	22 (16-44)	35 (21-48)	24 (16-44)	21 (16-33)	-	24 (16-51)
**T_{G0} (h)**	-	-	254 (81-439)	297 (168-392)	295 (139-439)	245 (107-422)	-	288 (125-438)
**T_N (h)**	-	-	16 (6-62)	16 (6-30)	26 (7-64)	14 (6-38)	-	23 (6-55)
**T_A (h)**	-	-	10 (1-25)	9 (1-22)	11 (1-23)	11 (1-24)	-	12 (1-25)
**N_{LIMP} (no.)**	-	-	8 (3-18)	10 (6-18)	9 (4-17)	7 (3-11)	-	9 (4-18)
**α (Gy^{-1})**	-	-	0.077 (0.046-0.265)	0.410 (0.145-0.688)	0.175 (0.052-0.635)	0.396 (0.121-0.679)	-	0.109 (0.045-0.433)
**OER**	-	-	2.24 (1.5-3)	2.00 (1.5-3)	2.19 (1.5-3)	2.11 (1.5-3)	-	2.25 (1.5-3)
**R_A (h^{-1})**	-	-	0.0019 (0.001-0.007)	0.0015 (0.001-0.004)	0.0014 (0.001-0.004)	0.0013 (0.001-0.003)	-	0.0017 (0.001-0.004)
**R_{NDiff} (h^{-1})**	-	-	0.0019 ((2.8E-06)-0.0070)	0.00057 ((3.1E-05)-0.0011)	0.00099 ((1.1E-06)-0.0042)	0.0029 ((5.9E-05)-0.0065)	-	0.001((1.6E-05)-0.0049)
**R_{ADiff} (h^{-1})**	-	-	0.0022 (0.001-0.007)	0.0012 (0.001-0.002)	0.0014 (0.001-0.004)	0.0017 (0.001-0.005)	-	0.0015 (0.001-0.005)
**P_{G0toG1} (h^{-1})**	-	-	0.035 (0.01-0.06)	0.033 (0.01-0.06)	0.038 (0.01-0.06)	0.036 (0.01-0.06)	-	0.035 (0.01-0.06)
**P_{sleep}**	-	-	0.301 (0.154-0.410)	0.348 (0.315-0.398)	0.313 (0.213-0.383)	0.280 (0.185-0.368)	-	0.311 (0.182-0.381)
**P_{sym}**	-	-	0.485 (0.2-0.66)	0.599 (0.52-0.66)	0.540 (0.32-0.66)	0.424 (0.24-0.64)	-	0.533 (0.26-0.66)

Supplementary Table S7. *Tumour profile GF=10%, HF=30%, DF=5%, CKR=0.1. Mean value and range of values of each CERONCO parameter. For the definition of CERONCO parameters see Table1 of main article. For the computation of CERONCO parameter values based on the user-defined tumour features see Supplementary Table S2. The β parameter of the LQ model is chosen so that α/β=10Gy in all simulations. The sublethal damage repair half-time* $T_{1/2}$ *is taken equal to 1.5Gy.* T_d *values displayed as: value resulting from the mean value parameter set (range of* T_d *in all solutions).*

**GF 10% HF 30% DF 5%, CKR=0.1 (For each parameter: mean value (range))**								
**Patient**	**50**	**55**	**68**	**71**	**77**	**86**	**88**	**95**
**No.of solutions**	**0**	**0**	**1174**	**18**	**67**	**28**	**0**	**110**
**T_d (days)**	**-**	**-**	**102 (20-4000)**	**318 (50-3000)**	**25 (20-50)**	**166 (50-4000)**		**23 (20-80)**
**T_c (h)**	-	-	22 (16-48)	35 (22-46)	24 (16-38)	21 (16-30)	-	22 (16-41)
**T_{G0} (h)**	-	-	255 (84-439)	286 (164-389)	320 (171-432)	249 (115-396)	-	296 (143-439)
**T_N (h)**	-	-	16 (6-67)	12 (6-23)	26 (7-65)	12 (6-36)	-	23 (6-74)
**T_A (h)**	-	-	10 (1-25)	11 (1-23)	12 (1-24)	11 (1-23)	-	12 (1-25)
**N_{LIMP} (no.)**	-	-	8 (3-18)	9 (6-13)	9 (4-18)	9 (4-16)	-	9 (4-17)
**α (Gy^{-1})**	-	-	0.072 (0.040-0.304)	0.342 (0.123-0.652)	0.195 (0.061-0.668)	0.335 (0.093-0.697)	-	0.101 (0.038-0.390)
**OER**	-	-	2.26 (1.5-3)	2.08 (1.5-3)	2.36 (1.5-3)	2.20 (1.5-3)	-	2.18 (1.5-3)
**R_A (h^{-1})**	-	-	0.0019 (0.001-0.006)	0.0014 (0.001-0.003)	0.0017 (0.001-0.004)	0.0019 (0.001-0.004)	-	0.0017 (0.001-0.005)
**R_{NDiff} (h^{-1})**	-	-	0.0019 ((3.9E-08)-0.0074)	0.00064 ((8.7E-06)-0.001)	0.0010 ((8.6E-06)-0.0045)	0.0025 ((5.6E-05)-0.0053)	-	0.0011 ((9.6E-06)-0.0041)
**R_{ADiff} (h^{-1})**	-	-	0.0021 (0.001-0.007)	0.0012 (0.001-0.002)	0.0014 (0.001-0.003)	0.0019 (0.001-0.004)	-	0.0015 (0.001-0.005)
**P_{G0toG1} (h^{-1})**	-	-	0.035 (0.01-0.06)	0.037 (0.01-0.06)	0.036 (0.01-0.06)	0.032 (0.01-0.06)	-	0.035 (0.01-0.06)
**P_{sleep}**	-	-	0.301 (0.153-0.408)	0.351 (0.315-0.391)	0.304 (0.203-0.390)	0.293 (0.168-0.368)	-	0.305 (0.211-0.379)
**P_{sym}**	-	-	0.485 (0.2-0.66)	0.602 (0.52-0.66)	0.524 (0.3-0.66)	0.465 (0.22-0.64)	-	0.524 (0.3-0.66)

Supplementary Table S8. *Tumour profile GF=10%, HF=30%, DF=5%, CKR=0.2. Mean value and range of values of each CERONCO parameter. For the definition of CERONCO parameters see Table1 of main article. For the computation of CERONCO parameter values based on the user-defined tumour features see Supplementary Table S2. The β parameter of the LQ model is chosen so that α/β=10Gy in all simulations The sublethal damage repair half-time $T_{1/2}$ is taken equal to 1.5Gy. T_d values displayed as: value resulting from the mean value parameter set (range of T_d in all solutions).*

**GF 10% HF 30% DF 5%, CKR=0.2 (For each parameter: mean value (range))**								
**Patient**	**50**	**55**	**68**	**71**	**77**	**86**	**88**	**95**
**No. of solutions**	**0**	**0**	**1583**	**268**	**39**	**1447**	**0**	**68**
**T_d (days)**	**-**	**-**	**99 (20-4000)**	**185 (20-3000)**	**22 (20-30)**	**83 (20-4000)**		**20 (20-30)**
**T_c (h)**	-	-	22 (16-46)	34 (17-50)	23 (16-29)	20 (16-36)	-	21 (16-34)
**T_{G0} (h)**	-	-	252 (81-439)	293 (116-438)	314 (150-435)	255 (81-439)	-	328 (144-437)
**T_N (h)**	-	-	16 (6-57)	13 (6-34)	25 (7-44)	14 (6-62)	-	24 (6-52)
**T_A (h)**	-	-	10 (1-25)	10 (1-24)	12 (1-25)	10 (1-25)	-	12 (1-25)
**N_{LIMP} (no.)**	-	-	8 (3-18)	10 (5-18)	9 (5-17)	7 (3-18)	-	8 (3-13)
**α (Gy^{-1})**	-	-	0.061 (0.024-0.678)	0.407 (0.067-0.697)	0.189 (0.047-0.675)	0.402 (0.054-0.699)	-	0.086 (0.031-0.390)
**OER**	-	-	2.24 (1.5-3)	2.24 (1.5-3)	2.14 (1.5-3)	2.24 (1.5-3)	-	2.35 (1.5-3)
**R_A (h^{-1})**	-	-	0.0019 (0.001-0.007)	0.0014 (0.001-0.004)	0.0016 (0.001-0.004)	0.0019 (0.001-0.007)	-	0.0016 (0.001-0.005)
**R_{NDiff} (h^{-1})**	-	-	0.0018((2.9E-06)-0.0071)	0.00069 ((1.4E-06)-0.002)	0.00086 ((4.2E-07)-0.0029)	0.0022 ((4.4E-06)-0.0069)	-	0.0013 ((8.6E-06)-0.0049)
**R_{ADiff} (h^{-1})**	-	-	0.0019 (0.001-0.008)	0.0011 (0.001-0.002)	0.0016 (0.001-0.004)	0.0023 (0.001-0.008)	-	0.0015 (0.001-0.003)
**P_{G0toG1} (h^{-1})**	-	-	0.035 (0.01-0.06)	0.034 (0.01-0.06)	0.035 (0.01-0.06)	0.035 (0.01-0.06)	-	0.037 (0.01-0.06)
**P_{sleep}**	-	-	0.302 (0.153-0.410)	0.344 (0.267-0.398)	0.304 (0.200-0.374)	0.288 (0.154-0.410)	-	0.285 (0.191-0.371)
**P_{sym}**	-	-	0.489 (0.2-0.66)	0.589 (0.44-0.66)	0.526 (0.3-0.66)	0.457 (0.2-0.66)	-	0.483 (0.28-0.66)

Supplementary Table S9. *Tumour profile GF=10%, HF=30%, DF=5%, CKR=0.3. Mean value and range of values of each CERONCO parameter. For the definition of CERONCO parameters see Table1 of main article. For the computation of CERONCO parameter values based on the user-defined tumour features see Supplementary Table S2. The β parameter of the LQ model is chosen so that α/β=10Gy in all simulations. The sublethal damage repair half-time $T_{1/2}$ is taken equal to 1.5Gy. Td values displayed as: value resulting from the mean value parameter set (range of Td in all solutions).*

**GF 10% HF 30% DF 5%, CKR=0.3 (For each parameter: mean value (range))**								
**Patient**	**50**	**55**	**68**	**71**	**77**	**86**	**88**	**95**
**No. of solutions**	**360**	**5**	**1536**	**444**	**20**	**1154**	**0**	**26**
**T_d (days)**	**98 (20-4000)**	**450 (350-1000)**	**97 (20-4000)**	**154 (20-4000)**	**20 ((20)**	**79 (20-4000)**		**20 (20)**
**T_c (h)**	18 (16-25)	39 (39-41)	22 (16-45)	34 (16-49)	23 (17-28)	20 (16-35)	-	24 (19-28)
**T_{G0} (h)**	254 (92-438)	261 (246-290)	253 (84-439)	289 (97-439)	313 (144-427)	253 (85-439)	-	342 (189-437)
**T_N (h)**	12 (6-35)	22 (19-32)	16 (6-66)	13 (6-32)	29 (10-52)	14 (6-60)	-	29 (8-52)
**T_A (h)**	9 (1-25)	21 (11-25)	10 (1-25)	10 (1-24)	12 (2-25)	10 (1-25)	-	12 (2-25)
**N_{LIMP} (no.)**	6 (3-16)	10 (10-11)	8 (3-18)	10 (5-18)	8 (6-13)	7(3-18)	-	8 (5-12)
**α (Gy^{-1})**	0.007 (0.0-0.567)	0.510 (0.367-0.567)	0.042 (0.008-0.619)	0.469 (0.060-0.619)	0.182 (0.035-0.505)	0.335 (0.052-0.567)	-	0.106 (0.025-0.390)
**OER**	2.21 (1.5-3)	2.20 (1.5-3)	2.25 (1.5-3)	2.19 (1.5-3)	2.33 (1.5-3)	2.26 (1.5-3)	-	2.40 (1.5-3)
**R_A (h^{-1})**	0.0018 (0.001-0.006)	0.001 (0.001)	0.0019 (0.001-0.007)	0.0014 (0.001-0.005)	0.0012 (0.001-0.003)	0.0019 (0.001-0.007)	-	0.0012 (0.001-0.003)
**R_{NDiff} (h^{-1})**	0.0028 ((6.7E-06)-0.0068)	0.00046 (0.0004-0.0005)	0.0018 ((1.3E-06)-0.0073)	0.00068 ((1.2E-06)-0.002)	0.00077 ((8.6E-06)-0.0034)	0.0023 ((6.8E-07)-0.0075)	-	0.00082 ((8.6E-06)-0.0034)
**R_{ADiff} (h^{-1})**	0.0027 (0.001-0.007)	0.001 (0.001)	0.0020 (0.001-0.008)	0.0011 (0.001-0.002)	0.0014 (0.001-0.003)	0.0022 (0.001-0.007)	-	0.0013 (0.001-0.003)
**P_{G0toG1} (h^{-1})**	0.035 (0.01-0.06)	0.04 (0.02-0.05)	0.034 (0.01-0.06)	0.035 (0.01-0.06)	0.035 (0.34-0.64)	0.0.35 (0.01-0.06)	-	0.036 (0.01-0.06)
**P_{sleep}**	0.263 (0.165-0.378)	0.378 (0.369-0.385)	0.304 (0.154-0.402)	0.347 (0.266-0.398)	0.300 (0.219-0.371)	0.288 (0.153-0.406)	-	0.293 (0.219-0.357)
**P_{sym}**	0.399 (0.22-0.64)	0.644 (0.62-0.66)	0.494 (0.2-0.66)	0.594 (0.44-0.66)	0.509 (0.34-0.64)	0.454 (0.2-0.66)	-	0.498 (0.34-0.66)

Supplementary Table S10. *Tumour profile GF=10%, HF=30%, DF=5%, CKR=0.4. Mean value and range of values of each CERONCO parameter. For the definition of CERONCO parameters see Table1 of main article. For the computation of CERONCO parameter values based on the user-defined tumour features see Supplementary Table S2. The β parameter of the LQ model is chosen so that α/β=10Gy in all simulations. The sublethal damage repair half-time $T_{1/2}$ is taken equal to 1.5Gy. Td values displayed as: value resulting from the mean value parameter set (range of Td in all solutions).*

GF 10% HF 30% DF 5%, CKR=0.4 (For each parameter: mean value (range))								
**Patient**	**50**	**55**	**68**	**71**	**77**	**86**	**88**	**95**
**No. of solutions**	**31**	**0**	**338**	**423**	**0**	**842**	**0**	**0**
**T_d (days)**	**28 (20-80)**	**-**	**42 (20-1000)**	**175 (20-4000)**	**-**	**87 (20-4000)**	**-**	**-**
**T_c (h)**	19 (16-28)	-	21 (16-38)	34 (16-50)	-	20 (16-34)	-	-
**T_{G0} (h)**	277 (88-439)	-	255 (92-439)	300 (117-437)	-	248 (82-439)	-	-
**T_N (h)**	18 (6-47)	-	16 (6-51)	14 (6-34)	-	14 (6-61)	-	-
**T_A (h)**	8 (1-20)	-	11 (1-25)	9 (1-25)	-	10 (1-25)	-	-
**N_{LIMP} (no.)**	7 (3-14)	-	8 (3-18)	10 (4-18)	-	7 (3-18)	-	-
**α (Gy^{-1})**	0.057 (0-0.431)	-	0.062 (0-0.471)	0.374 (0.056-0.471)	-	0.254 (0.050-0.431)	-	-
**OER**	2.18 (1.5-3)	-	2.25 (1.5-3)	2.23 (1.5-3)	-	2.25 (1.5-3)	-	-
**R_A (h^{-1})**	0.0013 (0.001-0.003)	-	0.0018 (0.001-0.007)	0.0014 (0.001-0.006)	-	0.0019 (0.001-0.006)	-	-
**R_{NDiff} (h^{-1})**	0.0020 ((9.1E-06)-0.0058)	-	0.0017 ((9.1E-06)-0.0065)	0.00065 ((1.2E-06)-0.001)	-	0.0021 ((2.2E-06)-0.0073)	-	-
**R_{ADiff} (h^{-1})**	0.0027 (0.001-0.006)	-	0.0018 (0.001-0.007)	0.0011 (0.001-0.002)	-	0.0023 (0.001-0.008)	-	-
**P_{G0toG1} (h^{-1})**	0.037 (0.01-0.06)	-	0.035 (0.01-0.06)	0.035 (0.01-0.06)	-	0.036 (0.01-0.06)	-	-
**P_{sleep}**	0.265 (0.184-0.394)	-	0.303 (0.174-0.401)	0.344 (0.266-0.402)	-	0.291 (0.156-0.406)	-	-
**P_{sym}**	0.409 (0.26-0.64)	-	0.500 (0.24-0.66)	0.590 (0.44-0.66)	-	0.461 (0.2-0.66)	-	-

Supplementary Table S11. *Tumour profile GF=10%, HF=30%, DF=5%, CKR=0.5. Mean value and range of values of each CERONCO parameter. For the definition of CERONCO parameters see Table1 of main article. For the computation of CERONCO parameter values based on the user-defined tumour features see Supplementary Table S2. The β parameter of the LQ model is chosen so that α/β=10Gy in all simulations. The sublethal damage repair half-time $T_{1/2}$ is taken equal to 1.5Gy. Td values displayed as: value resulting from the mean value parameter set (range of Td in all solutions).*

**GF 10% HF 30% DF 5%, CKR=0.5 (For each parameter: mean value (range))**								
**Patient**	**50**	**55**	**68**	**71**	**77**	**86**	**88**	**95**
**No. of solutions**	**17**	**0**	**123**	**110**	**0**	**603**	**0**	**0**
**T_d (days)**	**20 (20-30)**	**-**	**28 (20-600)**	**40 (20-150)**	**-**	**84 (20-4000)**	**-**	**-**
**T_c (h)**	17 (16-25)	-	20 (16-46)	26 (16-41)	-	20 (16-34)	-	-
**T_{G0} (h)**	286 (172-438)	-	235 (91-437)	273 (108-438)	-	251 (86-436)	-	-
**T_N (h)**	16 (6-41)	-	16 (6-52)	12 (6-23)	-	14 (6-57)	-	-
**T_A (h)**	11 (1-23)	-	11 (1-25)	9 (1-23)	-	10 (1-25)	-	-
**N_{LIMP} (no.)**	6 (3-10)	-	9 (3-16)	9 (5-18)	-	7 (3-18)	-	-
**α (Gy^{-1})**	0.032 (0.002-0.326)	-	0.083 (0-0.357)	0.315 (0.066-0.357)	-	0.197 (0.050-0.326)	-	-
**OER**	2.21 (1.5-3)	-	2.20 (1.5-3)	2.28 (1.5-3)	-	2.22 (1.5-3)	-	-
**R_A (h^{-1})**	0.0012 (0.001-0.002)	-	0.0018 (0.001-0.005)	0.0017 (0.001-0.004)	-	0.0019 (0.001-0.007)	-	-
**R_{NDiff} (h^{-1})**	0.0024 ((7.8E-06)-0.0049)	-	0.0015 ((8.6E-06)-0.0058)	0.00079 ((5.4E-09)-0.002)	-	0.0022 ((7.7E-07)-0.0075)	-	-
**R_{ADiff} (h^{-1})**	0.0022 (0.001-0.005)	-	0.0016 (0.001-0.004)	0.0012 (0.001-0.002)	-	0.0023 (0.001-0.007)	-	-
**P_{G0toG1} (h^{-1})**	0.029 (0.01-0.06)	-	0.037 (0.01-0.06)	0.036 (0.01-0.06)	-	0.035 (0.01-0.06)	-	-
**P_{sleep}**	0.251 (0.201-0.358)	-	0.312 (0.183-0.399)	0.330 (0.267-0.389)	-	0.287 (0.157-0.401)	-	-
**P_{sym}**	0.392 (0.3-0.62)	-	0.523 (0.26-0.66)	0.572 (0.44-0.66)	-	0.453 (0.2-0.66)	-	-

Supplementary Table S12. *Tumour profile GF=10%, HF=30%, DF=5%, CKR: 0.0-0.5. Range of mean values of each CERONCO parameter (from Supplementary Tables S5-S10). For the definition of CERONCO parameters see Table1 of main article. For the computation of CERONCO parameter values based on the user-defined tumour features see Supplementary Table S2. The β parameter of the LQ model is chosen so that α/β=10Gy in all simulations. The sublethal damage repair half-time $T_{1/2}$ is taken equal to 1.5Gy. Td values represent the volume doubling time(s) of the virtual tumour(s) resulting from the mean value parameter set(s).*

**GF 10% HF 30% DF 5%, CKR 0-0.5**<br>**Range of mean values of CERONCO parameters in the mean value sets, CKR 0-0.5**								
**Patient**	**50**	**55**	**68**	**71**	**77**	**86**	**88**	**95**
**T_d (days)**	**20-98**	**450**	**28-127**	**40-318**	**20-36**	**79-166**	**-**	**20-44**
**T_c (h)**	17-19	39	20-22	26-35	23-26	20-21	-	21-27
**T_{G0} (h)**	254-286	261	235-255	273-300	295-320	245-266	-	288-342
**T_N (h)**	12-18	22	16	12-16	25-29	12-15	-	23-29
**T_A (h)**	8-11	21	10-11	9-11	11-12	10-11	-	12
**N_{LIMP} (no.)**	6-7	10	8-9	9-11	8-9	7-9	-	8-9
**α (Gy^{-1})**	0.007-0.057	0.510	0.042-0.084	0.315-0.469	0.175-0.204	0.197-0.402	-	0.086-0.109
**OER**	2.18-2.21	2.20	2.20-2.26	2-2.28	2.12-2.36	2.11-2.26	-	2.18-2.40
**R_A (h^{-1})**	0.001-0.002	0.001	0.002	0.001-0.002	0.001-0.002	0.0013-0.0019	-	0.001-0.002
**R_{NDiff} (h^{-1})**	0.002-0.003	0.0005	0.002	0.0006-0.002	0.001	0.0020-0.0029	-	0.001
**R_{ADiff} (h^{-1})**	0.001-0.003	0.001	0.002	0.001	0.001	0.0017-0.0023	-	0.001-0.002
**P_{G0toG1} (h^{-1})**	0.029-0.037	0.040	0.034-0.037	0.033-0.037	0.035-0.038	0.032-0.038	-	0.035-0.038
**P_{sleep}**	0.251-0.265	0.378	0.301-0.312	0.330-0.351	0.300-0.314	0.280-0.293	-	0.285-0.318
**P_{sym}**	0.392-0.409	0.644	0.485-0.523	0.572-0.602	0.509-0.540	0.424-0.465	-	0.483-0.542

Supplementary Table S13. *Tumour profile GF=60%, HF=30%, DF=5%, CKR: 0.0-0.5 Range of CERONCO parameter values from all identified solutions. For the definition of CERONCO parameters see Table1 of main article. For the computation of CERONCO parameter values based on the user-defined tumour features see Supplementary Table S2. The β parameter of the LQ model is chosen so that α/β=10Gy in all simulations. The sublethal damage repair half-time $T_{1/2}$ is taken equal to 1.5Gy. Td values represent the volume doubling time(s) of the virtual tumour(s) resulting from the mean value parameter set(s).*

**GF 60% HF 30% DF 5%, CKR 0-0.5<br>Range of CERONCO parameter values from all solutions**								
**Patient**	**50**	**55**	**68**	**71**	**77**	**86**	**88**	**95**
**No. of solutions**	**289**	**7**	**5353**	**0**	**496**	**10775**	**275**	**153**
**T_d (days)**	**20-250**	**20**	**20-1500**	**-**	**20-50**	**20-2000**	**20-200**	**20-50**
**T_c (h)**	27-70	38-50	26-70	-	26-70	25-70	39-70	35-70
**T_{G0} (h)**	67-433	61-111	61-439	-	74-439	61-439	113-438	132-439
**T_N (h)**	6-28	6-7	6-25	-	6-28	6-37	6-48	6-45
**T_A (h)**	1-16	1-6	1-19	-	1-23	1-19	1-25	1-19
**N_{LIMP} (no.)**	5-18	12-15	4-18	-	5-18	4-18	5-18	5-18
**α (Gy^{-1})**	0.004-0.042	0.002-0.037	0.000-0.149	-	0.038-0.103	0.050-0.699	0.050-0.165	0.036-0.071
**OER**	1.5-3	1.5-3	1.5-3	-	1.5-3	1.5-3	1.5-3	1.5-3
**R_A (h^{-1})**	0.001-0.01	0.001-0.002	0.001-0.01	-	0.001-0.008	0.001-0.011	0.001-0.004	0.001-0.005
**R_{NDiff} (h^{-1})**	0.004-0.236	0.068-0.141	0.0015-0.249	-	0.006-0.242	(1.7E-05)-0.257	0.002-0.237	0.002-0.209
**R_{ADiff} (h^{-1})**	0.001-0.1	0.019-0.071	0.001-0.1	-	0.001-0.1	0.001-0.1	0.001-0.1	0.001-0.1
**P_{G0toG1} (h^{-1})**	0.01-0.06	0.01-0.05	0.01-0.06	-	0.01-0.06	0.01-0.06	0.01-0.06	0.01-0.06
**P_{sleep}**	0.125-0.334	0.256-0.328	0.110-0.369	-	0.125-0.318	0.108-0.377	0.128-0.273	0.119-0.270
**P_{sym}**	0.24-0.6	0.54-0.66	0.2-0.66	-	0.26-0.66	0.2-0.66	0.24-0.66	0.24-0.66

Supplementary Table S14. *Tumour profile GF=60%, HF=30%, DF=5%, CKR=0.0. Mean value and range of values of each CERONCO parameter. For the definition of CERONCO parameters see Table1 of main article. For the computation of CERONCO parameter values based on the user-defined tumour features see Supplementary Table S2. The* β *parameter of the LQ model is chosen so that* $\alpha/\beta=10Gy$ *in all simulations. The sublethal damage repair half-time* $T_{1/2}$ *is taken equal to 1.5Gy.* T_d *values displayed as: value resulting from the mean value parameter set (range of* T_d *in all solutions).*

**GF 60% HF 30% DF 5%, CKR=0.0 (For each parameter: mean value (range))**									
**Patient**	**50**	**55**	**68**	**71**	**77**	**86**	**88**	**95**	
**No. of solutions**	**0**	**0**	**1285**	**0**	**147**	**1779**	**69**	**31**	
**T_d (days)**	**-**	**-**	**100 (20-1500)**	**-**	**28 (20-50)**	**66 (20-1000)**	**20 (20-200)**	**33 (20-50)**	
**T_c (h)**	-	-	56 (28-70)	-	55 (26-70)	57 (25-70)	60 (39-70)	62 (54-70)	
**T_{G0} (h)**	-	-	175 (61-439)	-	197 (74-404)	197 (62-439)	364 (113-431)	268 (132-438)	
**T_N (h)**	-	-	8 (6-25)	-	8 (6-28)	8 (6-37)	11 (6-48)	8 (6-23)	
**T_A (h)**	-	-	3 (1-17)	-	4 (1-14)	4 (1-19)	6 (1-16)	5 (1-10)	
**N_{LIMP} (no.)**	-	-	11 (4-18)	-	11 (5-18)	11 (4-18)	12 (6-18)	9 (5-18)	
**α (Gy^{-1})**	-	-	0.064 (0.040-0.141)	-	0.064 (0.043-0.103)	0.280 (0.055-0.499)	0.109 (0.050-0.165)	0.048 (0.039-0.063	
**OER**	-	-	2.24 (1.5-3)	-	2.26 (1.5-3)	2.27 (1.5-3)	2.26 (1.5-3)	2.33 (1.5-3)	
**R_A (h^{-1})**	-	-	0.0025 (0.001-0.01)	-	0.0021 (0.001-0.008)	0.0024 (0.001-0.011)	0.0016 (0.001-0.004)	0.0015 (0.001-0.003)	
**R_{NDiff} (h^{-1})**	-	-	0.103 (0.003-0.246)	-	0.106 (0.006-0.215)	0.105 ((1.69E-05)-0.257)	0.093 (0.002-0.237)	0.111 (0.009-0.209)	
**R_{ADiff} (h^{-1})**	-	-	0.051 (0.001-0.1)	-	0.049 (0.001-0.1)	0.050 (0.001-0.1)	0.041 (0.001-0.1)	0.053 (0.005-0.094)	
**P_{G0toG1} (h^{-1})**	-	-	0.036 (0.01-0.06)	-	0.036 (0.01-0.06)	0.035 (0.01-0.06)	0.038 (0.01-0.06)	0.040 (0.01-0.06)	
**P_{sleep}**	-	-	0.236 (0.113-0.369)	-	0.218 (0.127-0.318)	0.225 (0.110-0.372)	0.179 (0.132-0.273)	0.193 (0.119-0.270)	
**P_{sym}**	-	-	0.517 (0.2-0.66)	-	0.517 (0.26-0.66)	0.508 (0.2-0.66)	0.528 (0.24-0.66)	0.429 (0.24-0.62)	

Supplementary Table S15. *Tumour profile GF=60%, HF=30%, DF=5%, CKR=0.05. Mean value and range of values of each CERONCO parameter. For the definition of CERONCO parameters see Table1 of main article. For the computation of CERONCO parameter values based on the user-defined tumour features see Supplementary Table S2. The β parameter of the LQ model is chosen so that α/β=10Gy in all simulations. The sublethal damage repair half-time* $T_{1/2}$ *is taken equal to 1.5Gy.Td values displayed as: value resulting from the mean value parameter set (range of Td in all solutions).*

**GF 60% HF 30% DF 5%, CKR=0.05 (For each parameter: mean value (range))**								
**Patient**	**50**	**55**	**68**	**71**	**77**	**86**	**88**	**95**
**No. of solutions**	**0**	**0**	**949**	**0**	**110**	**1818**	**61**	**25**
**T_d (days)**	**-**	**-**	**68 (20-400)**	**-**	**23 (20-50)**	**61 (20-1000)**	**20 (20-150)**	**26 (20-45)**
**T_c (h)**	-	-	55 (26-70)	-	54 (26-70)	57 (25-70)	59 (39-70)	60 (45-69)
**T_{G0} (h)**	-	-	180 (66-438)	-	226 (81-439)	198 (62-439)	374 (179-434)	304 (140-434)
**T_N (h)**	-	-	8 (6-20)	-	8 (6-24)	8 (6-37)	9 (6-25)	11 (6-45)
**T_A (h)**	-	-	3 (1-14)	-	4 (1-14)	4 (1-19)	7 (1-20)	4 (1-9)
**N_{LIMP} (no.)**	-	-	11 (5-18)	-	11 (5-18)	11 (4-18)	11 (5-18)	9 (5-16)
**α (Gy^{-1})**	-	-	0.062 (0.036-0.149)	-	0.064 (0.042-0.103)	0.278 (0.062-0.499)	0.107 (0.058-0.168)	0.047 (0.037-0.059)
**OER**	-	-	2.25 (1.5-3)	-	2.27 (1.5-3)	2.27 (1.5-3)	2.21 (1.5-3)	2.31 (1.5-3)
**R_A (h^{-1})**	-	-	0.0025 (0.001-0.1)	-	0.0022 (0.001-0.008)	0.0024 (0.001-0.011)	0.0013 (0.001-0.003)	0.0018 (0.001-0.003)
**R_{NDiff} (h^{-1})**	-	-	0.107 (0.002-0.249)	-	0.115 (0.021-0.242)	0.106 ((1.7E-05)-0.253)	0.101 (0.005-0.237)	0.079 (0.002-0.163)
**R_{ADiff} (h^{-1})**	-	-	0.050 (0.001-0.1)	-	0.047 (0.003-0.1)	0.050 (0.001-0.1)	0.046 (0.001-0.1)	0.047 (0.002-0.099)
**P_{G0toG1} (h^{-1})**	-	-	0.034 (0.01-0.06)	-	0.035 (0.01-0.06)	0.035 (0.01-0.06)	0.035 (0.01-0.06)	0.035 (0.01-0.06)
**P_{sleep}**	-	-	0.230 (0.110-0.366)	-	0.206 (0.125-0.303)	0.225 (0.110-0.372)	0.172 (0.128-0.236)	0.175 (0.127-0.260)
**P_{sym}**	-	-	0.516 (0.2-0.66)	-	0.509 (0.26-0.66)	0.508 (0.2-0.66)	0.496 (0.24-0.66)	0.430 (0.28-0.64)

Supplementary Table S16. *Tumour profile GF=60%, HF=30%, DF=5%, CKR=0.1. Mean value and range of values of each CERONCO parameter. For the definition of CERONCO parameters see Table1 of main article. For the computation of CERONCO parameter values based on the user-defined tumour features see Supplementary Table S2. The β parameter of the LQ model is chosen so that α/β=10Gy in all simulations. The sublethal damage repair half-time $T_{1/2}$ is taken equal to 1.5Gy.Td values displayed as: value resulting from the mean value parameter set (range of Td in all solutions).*

**GF 60% HF 30% DF 5%, CKR=0.1 (For each parameter: mean value (range))**								
**Patient**	**50**	**55**	**68**	**71**	**77**	**86**	**88**	**95**
**No. of solutions**	**0**	**0**	**974**	**0**	**100**	**1813**	**47**	**26**
**T_d (days)**	-	-	**71 (20-400)**	**-**	**20 (20-35)**	**55 (20-1000)**	**20 (20-40)**	**22 (20-35)**
**T_c (h)**	-	-	55 (26-70)	-	52 (26-70)	56 (27-70)	61 (44-70)	61 (44-70)
**T_{G0} (h)**	-	-	182 (65-438)	-	216 (74-439)	197 (62-439)	386 (325-438)	292 (139-429)
**T_N (h)**	-	-	8 (6-19)	-	8 (6-25)	8 (6-28)	12 (6-28)	11 (6-28)
**T_A (h)**	-	-	3 (1-14)	-	4 (1-14)	4 (1-19)	6 (1-17)	5 (1-16)
**N_{LIMP} (no.)**	-	-	11 (5-18)	-	12 (5-18)	11 (5-18)	12 (7-18)	11 (5-16)
**α (Gy^{-1})**	-	-	0.054 (0.029-0.142)	-	0.065 (0.044-0.094)	0.377 (0.059-0.699)	0.104 (0.059-0.146)	0.049 (0.036-0.069)
**OER**	-	-	2.27 (1.5-3)	-	2.24 (1.5-3)	2.27 (1.5-3)	2.22 (1.5-3)	2.40 (1.5-3)
**R_A (h^{-1})**	-	-	0.0025 (0.001-0.01)	-	0.0024 (0.001-0.008)	0.002 (0.001-0.01)	0.0013 (0.001-0.003)	0.0016 (0.001-0.005)
**R_{NDiff} (h^{-1})**	-	-	0.107 (0.002-0.249)	-	0.102 (0.019-0.213)	0.105 (0.001-0.254)	0.079 (0.004-0.157)	0.098 (0.005-0.225)
**R_{ADiff} (h^{-1})**	-	-	0.050 (0.001-0.1)	-	0.051 (0.001-0.1)	0.050 (0.001-0.1)	0.051 (0.004-0.096)	0.054 (0.004-0.1)
**P_{G0toG1} (h^{-1})**	-	-	0.035 (0.01-0.06)	-	0.035 (0.01-0.06)	0.035 (0.01-0.06)	0.034 (0.01-0.06)	0.044 (0.01-0.06)
**P_{sleep}**	-	-	0.230 (0.110-0.367)	-	0.213 (0.125-0.303)	0.226 (0.108-0.376)	0.177 (0.148-0.205)	0.191 (0.128-0.256)
**P_{sym}**	-	-	0.515 (0.2-0.66)	-	0.551 (0.28-0.66)	0.512 (0.2-0.66)	0.529 (0.34-0.66)	0.474 (0.28-0.62)

Supplementary Table S17. Tumour profile GF=60%, HF=30%, DF=5%, CKR=0.2. Mean value and range of values of each CERONCO parameter. *For the definition of CERONCO parameters see Table1 of main article. For the computation of CERONCO parameter values based on the user-defined tumour features see Supplementary Table S2. The β parameter of the LQ model is chosen so that α/β=10Gy in all simulation. The sublethal damage repair half-time* $T_{1/2}$ *is taken equal to 1.5Gy. Td values displayed as: value resulting from the mean value parameter set (range of Td in all solutions).*

.

**GF 60% HF 30% DF 5%, CKR=0.2<br>(For each parameter: mean value (range))**								
**Patient**	**50**	**55**	**68**	**71**	**77**	**86**	**88**	**95**
**No. of solutions**	**14**	**0**	**1007**	**0**	**58**	**1898**	**52**	**26**
**T_d (days)**	**189 (100-250)**	**-**	**71 (20-400)**	**-**	**20 (20)**	**50 (20-1000)**	**20 (20)**	**20 (20)**
**T_c (h)**	54 (37-66)	-	56 (27-70)	-	50 (33-68)	56 (26-70)	58 (42-70)	59 (47-70)
**T_{G0} (h)**	189 (79-326)	-	184 (66-438)	-	212 (74-430)	200 (62-439)	384 (316-436)	285 (142-413)
**T_N (h)**	6 (6-8)	-	8 (6-20)	-	8 (6-18)	8 (6-28)	12 (6-43)	10 (6-19)
**T_A (h)**	3 (1-7)	-	3 (1-19)	-	5 (1-15)	4 (1-19)	8 (1-25)	6 (1-19)
**N_{LIMP} (no.)**	9 (6-16)	-	11 (5-18)	-	12 (6-18)	11 (5-18)	12 (8-17)	12 (7-17)
**α (Gy^{-1})**	0.011 (0.009-0.015)	-	0.039 (0.014-0.127)	-	0.064 (0.044-0.086)	0.373 (0.050-0.699)	0.105 (0.054-0.142)	0.051 (0.040-0.057)
**OER**	2.18 (1.5-3)	-	2.26 (1.5-3)	-	2.21 (1.5-3)	2.27 (1.5-3)	2.24 (1.5-3)	2.22 (1.5-3)
**R_A (h^{-1})**	0.0026 (0.001-0.007)	-	0.0025 (0.001-0.01)	-	0.0017 (0.001-0.004)	0.002 (0.001-0.01)	0.001 (0.001-0.002)	0.0015 (0.001-0.003)
**R_{NDiff} (h^{-1})**	0.136 (0.049-0.226)	-	0.106 (0.0015-0.249)	-	0.119 (0.025-0.226)	0.106 (0.002-0.254)	0.092 (0.003-0.201)	0.093 (0.015-0.205)
**R_{ADiff} (h^{-1})**	0.047 (0.008-0.085)	-	0.049 (0.001-0.1)	-	0.047 (0.002-0.094)	0.050 (0.001-0.1)	0.040 (0.001-0.1)	0.045 (0.001-0.1)
**P_{G0toG1} (h^{-1})**	0.029 (0.01-0.06)	-	0.035 (0.01-0.06)	-	0.039 (0.01-0.06)	0.035 (0.01-0.06)	0.032 (0.01-0.06)	0.040 (0.01-0.06)
**P_{sleep}**	0.214 (0.145-0.318)	-	0.229 (0.108-0.366)	-	0.205 (0.146-0.293)	0.224 (0.110-0.372)	0.177 (0.145-0.201)	0.198 (0.144-0.269)
**P_{sym}**	0.450 (0.24-0.6)	-	0.514 (0.2-0.66)	-	0.526 (0.36-0.66)	0.511 (0.2-0.66)	0.548 (0.36-0.66)	0.527 (0.36-0.66)

Supplementary Table S18. Tumour profile GF=60%, HF=30%, DF=5%, CKR=0.3. Mean value and range of values of each CERONCO parameter. *For the definition of CERONCO parameters see Table1 of main article. For the computation of CERONCO parameter values based on the user-defined tumour features see Supplementary Table S2. The β parameter of the LQ model is chosen so that α/β=10Gy in all simulations. The sublethal damage repair half-time* $T_{1/2}$ *is taken equal to 1.5Gy. Td values displayed as: value resulting from the mean value parameter set (range of Td in all solutions).*

**GF 60% HF 30% DF 5%, CKR=0.3 (For each parameter: mean value (range))**								
**Patient**	**50**	**55**	**68**	**71**	**77**	**86**	**88**	**95**
**No. of solutions**	**128**	**0**	**826**	**0**	**65**	**1678**	**46**	**14**
**T_d (days)**	**51 (30-100)**		**55 (20-250)**	**-**	**20 (20)**	**52 (20-2000)**	**20 (20)**	**20 (20)**
**T_c (h)**	57 (27-70)	-	56 (27-70)	-	49 (35-63)	56 (27-70)	58 (42-70)	51 (40-63)
**T_{G0} (h)**	188 (67-433)	-	188 (66-438)	-	218 (117-428)	200 (62-439)	384 (333-436)	286 (183-426)
**T_N (h)**	8 (6-18)	-	8 (6-20)	-	8 (6-19)	8 (6-28)	13 (6-43)	8 (6-11)
**T_A (h)**	4 (1-10)	-	4 (1-19)	-	5 (1-20)	3 (1-19)	10 (1-25)	6 (1-17)
**N_{LIMP} (no.)**	11 (5-18)	-	11 (5-18)	-	13 (6-18)	11 (5-18)	13 (8-18)	12 (7-17)
**α (Gy^{-1})**	0.014 (0.004-0.024)	-	0.024 (0.001-0.100)	-	0.060 (0.043-0.075)	0.311 (0.050-0.637)	0.092 (0.064-0.121)	0.054 (0.049-0.059)
**OER**	2.29 (1.5-3)	-	2.26 (1.5-3)	-	2.34 (1.5-3)	2.26 (1.5-3)	2.34 (1.5-3)	2.39 (1.5-3)
**R_A (h^{-1})**	0.0023 (0.001-0.01)	-	0.0024 (0.001-0.01)	-	0.0017 (0.001-0.004)	0.0024 (0.001-0.1)	0.0010 (0.001-0.002)	0.0015 (0.001-0.002)
**R_{NDiff} (h^{-1})**	0.101 (0.004-0.236)	-	0.107 (0.004-0.242)	-	0.108 (0.019-0.237)	0.106 (0.002-0.254)	0.086 (0.003-0.201)	0.124 (0.022-0.180)
**R_{ADiff} (h^{-1})**	0.052 (0.001-0.099)	-	0.048 (0.001-0.1)	-	0.041 (0.001-0.096)	0.050 (0.001-0.1)	0.035 (0.001-0.1)	0.045 (0.002-0.098)
**P_{G0toG1} (h^{-1})**	0.035 (0.01-0.06)	-	0.034 (0.01-0.06)	-	0.041 (0.01-0.06)	0.035 (0.01-0.06)	0.033 (0.01-0.06)	0.037 (0.01-0.06)
**P_{sleep}**	0.227 (0.125-0.334)	-	0.227 (0.110-0.366)	-	0.207 (0.140-0.263)	0.224 (0.110-0.377)	0.182 (0.153-0.205)	0.183 (0.146-0.237)
**P_{sym}**	0.516 (0.24-0.66	-	0.513 (0.2-0.66)	-	0.571 (0.34-0.66)	0.510 (0.2-0.66)	0.579 (0.44-0.66)	0.514 (0.4-0.66)

Supplementary Table S19. *Tumour profile GF=60%, HF=30%, DF=5%, CKR=0.4. Mean value and range of values of each CERONCO parameter. For the definition of CERONCO parameters see Table1 of main article. For the computation of CERONCO parameter values based on the user-defined tumour features see Supplementary Table S2. The β parameter of the LQ model is chosen so that α/β=10Gy in all simulations. The sublethal damage repair half-time $T_{1/2}$ is taken equal to 1.5Gy. T_d values displayed as: value resulting from the mean value parameter set (range of T_d in all solutions).*

	**GF 60% HF 30% DF 5%, CKR=0.4<br>(For each parameter: mean value (range))**							
**Patient**	**50**	**55**	**68**	**71**	**77**	**86**	**88**	**95**
**No. of solutions**	**137**	**0**	**293**	**0**	**16**	**1110**	**-**	**23**
**T_d (days)**	**24 (20-35)**	**-**	**29 (20-40)**	**-**	**20 (20)**	**45 (20-1000)**	**-**	**20 (20)**
**T_c (h)**	58 (36-70)	-	55 (29-70)	-	52 (37-64)	56 (28-70)	-	53 (35-67)
**T_{G0} (h)**	217 (78-428)	-	190 (69-438)	-	262 (135-408)	203 (62-439)	-	331 (155-439)
**T_N (h)**	9 (6-28)	-	8 (6-24)	-	9 (6-14)	8 (6-25)	-	12 (6-27)
**T_A (h)**	4 (1-16)	-	4 (1-19)	-	7 (2-23)	4 (1-19)	-	5 (1-19)
**N_{LIMP} (no.)**	12 (5-18)	-	12 (5-18)	-	14 (8-18)	11 (5-18)	-	13 (7-17)
**α (Gy^{-1})**	0.016 (0.006-0.034)	-	0.020 (0-0.075)	-	0.048 (0.038-0.060)	0.245 (0.05-0.431)	-	0.054 (0.038-0.071)
**OER**	2.22 (1.5-3)	-	2.24 (1.5-3)	-	2.14 (1.5-3)	2.28 (1.5-3)	-	2.39 (1.5-3)
**R_A (h^{-1})**	0.0019 (0.001-0.005)	-	0.0022 (0.001-0.009)	-	0.0015 (0.001-0.003)	0.0023 (0.001-0.009)	-	0.0013 (0.001-0.004)
**R_{NDiff} (h^{-1})**	0.100 (0.005-0.215)	-	0.110 (0.010-0.242)	-	0.077 (0.015-0.220)	0.106 (0.002-0.253)	-	0.093 (0.019-0.182)
**R_{ADiff} (h^{-1})**	0.046 (0.001-0.1)	-	0.045 (0.001-0.1)	-	0.059 (0.005-0.097)	0.050 (0.001-0.1)	-	0.048 (0.008-0.091)
**P_{G0toG1} (h^{-1})**	0.037 (0.01-0.06)	-	0.035 (0.01-0.06)	-	0.041 (0.01-0.06)	0.035 (0.01-0.06)	-	0.033 (0.01-0.06)
**P_{sleep}**	0.217 (0.128-0.321)	-	0.223 (0.130-0.340)	-	0.204 (0.152-0.256)	0.222 (0.110-0.368)	-	0.184 (0.148-0.224)
**P_{sym}**	0.523 (0.28-0.66)	-	0.523 (0.28-0.66)	-	0.589 (0.48-0.66)	0.513 (0.2-0.66)	-	0.563 (0.44-0.66)

Supplementary Table S20. *Tumour profile GF=60%, HF=30%, DF=5%, CKR=0.5. Mean value and range of values of each CERONCO parameter. For the definition of CERONCO parameters see Table1 of main article. For the computation of CERONCO parameter values based on the user-defined tumour features see Supplementary Table S2. The* β *parameter of the LQ model is chosen so that* α/β*=10Gy in all simulations. The sublethal damage repair half-time* $T_{1/2}$ *is taken equal to 1.5Gy.* T_d *values displayed as: value resulting from the mean value parameter set (range of* T_d *in all solutions).*

**GF 60% HF 30% DF 5%, CKR=0.5 (For each parameter: mean value (range))**								
**Patient**	**50**	**55**	**68**	**71**	**77**	**86**	**88**	**95**
**No. of solutions**	**10**	**7**	**19**	**0**	**0**	**679**	**0**	**8**
**T_d (days)**	**20 (20)**	**20 (20)**	**20 (20)**	**-**	**-**	**46 (20-1000)**	**-**	**20 (20)**
**T_c (h)**	58 (43-67)	45 (38-50)	52 (30-70)	-	-	57 (26-70)	-	46 (40-51)
**T_{G0} (h)**	387 (313-433)	94 (61-111)	169 (99-258)	-	-	193 (61-439)	-	378 (338-425)
**T_N (h)**	12 (6-21)	6 (6-7)	7 (6-9)	-	-	8 (6-36)	-	9 (6-15)
**T_A (h)**	5 (1-10)	3 (1-6)	7 (1-17)	-	-	4 (1-19)	-	5 (1-9)
**N_{LIMP} (no.)**	11 (7-17)	14 (12-15)	13(8-18)	-	-	12 (4-18)	-	13 (9-17)
**α (Gy^{-1})**	0.036 (0.026-0.042)	0.014 (0.002-0.037)	0.012 (0.003-0.036)	-	-	0.168 (0.05-0.281)	-	0.054 (0.047-0.059)
**OER**	2.7 (1.5-3)	2.07 (1.5-3)	2.26 (1.5-3)	-	-	2.25 (1.5-3)	-	2.07 (1.5-3)
**R_A (h^{-1})**	0.0013 (0.001-0.002)	0.0014 (0.001-0.002)	0.0017 (0.001-0.005)	-	-	0.0023 (0.001-0.009)	-	0.0016 (0.001-0.002)
**R_{NDiff} (h^{-1})**	0.088 (0.030-0.202)	0.113 (0.068-0.141)	0.104 (0.039-0.164)	-	-	0.101 (0.001-0.219)	-	0.103 (0.034-0.180)
**R_{ADiff} (h^{-1})**	0.054 (0.007-0.082)	0.041 (0.019-0.071)	0.043 (0.001-0.096)	-	-	0.050 (0.001-0.1)	-	0.055 (0.011-0.099)
**P_{G0toG1} (h^{-1})**	0.036 (0.01-0.06)	0.034 (0.01-0.05)	0.031 (0.01-0.06)	-	-	0.035 (0.01-0.06)	-	0.031 (0.01-0.06)
**P_{sleep}**	0.172 (0.147-0.190)	0.271 (0.256-0.328)	0.227 (0.168-0.300)	-	-	0.229 (0.112-0.367)	-	0.171 (0.160-0.179)
**P_{sym}**	0.514 (0.38-0.62)	0.600 (0.54-0.66)	0.564 (0.44-0.66)	-	-	0.521 (0.2-0.66)	-	0.560 (0.52-0.62)

Supplementary Table S21. *Tumour profile GF=60%, HF=30%, DF=5%, CKR: 0.0-0.5. Range of mean values of each CERONCO parameter (from Supplementary Tables S5-S10). For the definition of CERONCO parameters see Table1 of main article. For the computation of CERONCO parameter values based on the user-defined tumour features see Supplementary Table S2. The β parameter of the LQ model is chosen so that α/β=10Gy in all simulations. The sublethal damage repair half-time $T_{1/2}$ is taken equal to 1.5Gy. Td values represent the volume doubling time(s) of the virtual tumour(s) resulting from the mean value parameter set(s).*

**GF 60% HF 30% DF 5%, CKR 0-0.5<br>Range of mean values of CERONCO parameters in the mean value sets, CKR 0-0.5**								
**Patient**	**50**	**55**	**68**	**71**	**77**	**86**	**88**	**95**
**T_d (days)**	**20-51**	**20**	**20-100**	**-**	**20-28**	**45-66**	**20**	**20-33**
**T_c (h)**	54-58	45	52-56	-	49-55	56-57	58-61	46-62
**T_{G0} (h)**	188-387	94	169-190	-	197-262	193-203	364-386	268-378
**T_N (h)**	6-12	6	7-8	-	8-9	8	9-13	8-12
**T_A (h)**	3-5	3	3-7	-	4-7	3-4	6-10	4-6
**N_{LIMP} (no.)**	9-12	14	11-13	-	11-14	11-12	11-13	9-13
**α (Gy^{-1})**	0.011-0.036	0.014	0.012-0.064	-	0.048-0.065	0.168-0.377	0.092-0.109	0.047-0.054
**OER**	2.18-2.7	2.07	2.24-2.27	-	2.14-2.34	2.25-2.28	2.21-2.34	2.07-2.40
**R_A (h^{-1})**	0.0013-0.0026	0.0014	0.0017-0.0025	-	0.0015-0.0024	0.002-0.0024	0.001-0.0016	0.0013-0.0018
**R_{NDiff} (h^{-1})**	0.088-0.136	0.113	0.103-0.110	-	0.077-0.119	0.101-0.106	0.079-0.101	0.079-0.124
**R_{ADiff} (h^{-1})**	0.046-0.054	0.041	0.043-0.051	-	0.041-0.059	0.050	0.035-0.051	0.045-0.055
**P_{G0toG1} (h^{-1})**	0.029-0.037	0.034	0.031-0.036	-	0.035-0.041	0.035	0.032-0.038	0.031-0.044
**P_{sleep}**	0.172-0.227	0.271	0.223-0.236	-	0.204-0.218	0.222-0.229	0.172-0.182	0.171-0.198
**P_{sym}**	0.450-0.523	0.600	0.513-0.564	-	0.509-0.589	0.508-0.521	0.496-0.579	0.429-0.563

SUPPLEMENTARY MATERIAL REFERENCES

1. Brenner, D. J., Dale, R., Orton, C., Fowler, J. Radiobiology of high dose-rate, low dose-rate, and pulsed dose-rate brachytherapy. In *Principles and Practice of Brachytherapy* (eds. Flynn, A. et al.). p. 189-204 (Arnold, 2001).

2. Pötter, R. et al. Recommendations from gynaecological (GYN) GEC ESTRO working group (II): concepts and terms in 3D image-based treatment planning in cervix cancer brachytherapy-3D dose volume parameters and aspects of 3D image-based anatomy, radiation physics, radiobiology. Radiother Oncol 78, 67–77 (2006).

3. Polo, A. Pulsed dose rate brachytherapy. Clin Transl Oncol 10, 324–333 (2008).

4. De Leeuw, A. A. C., Van de Kamer, J. B., Moerland, M. A., Philippens, M. E. P. & Jürgenliemk-Schulz, I.-M. The effect of alternative biological modelling parameters (α/β and half time of repair T1/2) on reported EQD2 values in the treatment of advanced cervical cancer. Radiotherapy and Oncology 101, 337–342 (2011).

5. Carlson, D. J., Stewart, R. D. & Semenenko, V. A. Effects of oxygen on intrinsic radiation sensitivity: A test of the relationship between aerobic and hypoxic linear-quadratic (LQ) model parameters. Med Phys 33, 3105–3115 (2006).

6. Tanderup, K. et al. 54 speaker EMBRACE UPDATE. Radiotherapy and Oncology 99, S22–S23 (2011).

7. Dimopoulos, J. C. A. et al. Recommendations from Gynaecological (GYN) GEC-ESTRO Working Group (IV): Basic principles and parameters for MR imaging within the frame of image based adaptive cervix cancer brachytherapy. Radiother Oncol 103, 113–122 (2012).

8. Kolokotroni, E. et al. In Silico Oncology: Quantification of the In Vivo Antitumor Efficacy of Cisplatin-Based Doublet Therapy in Non-Small Cell Lung Cancer (NSCLC) through a Multiscale Mechanistic Model. PLoS Comput. Biol. 12, e1005093 (2016).
